# The Murine Bladder Supports a Population of Stromal Sca-1^+^/CD34^+^/lin^-^ Mesenchymal Stem Cells

**DOI:** 10.1371/journal.pone.0141437

**Published:** 2015-11-05

**Authors:** Meredith A. Lilly, Natalie A. Kulkulka, Paula R. Firmiss, Michael J. Ross, Andrew S. Flum, Grace B. Delos Santos, Diana K. Bowen, Robert W. Dettman, Edward M. Gong

**Affiliations:** 1 Northwestern University, Feinberg School of Medicine, Department of Urology, 303 E. Chicago Ave., 16–703, Chicago, Illinois, 60611, United States of America; 2 Developmental Biology, Stanley Manne Children’s Research Institute, Anne and Robert H. Lurie Children’s Hospital of Chicago, 225 E. Chicago Ave. Box 225, Chicago, Illinois, 60611, United States of America; 3 Loyola University Health System, Department of Urology, 2160 S. First St., Maywood, Illinois, United States of America; Oklahoma University Health Sciences Center, UNITED STATES

## Abstract

Bladder fibrosis is an undesired end point of injury of obstruction and often renders the smooth muscle layer noncompliant. In many cases, the long-term effect of bladder fibrosis is renal failure. Despite our understanding of the progression of this disease, little is known about the cellular mechanisms that lead to a remodeled bladder wall. Resident stem (progenitor) cells have been identified in various organs such as the brain, heart and lung. These cells function normally during organ homeostasis, but become dysregulated after organ injury. Here, we aimed to characterize a mesenchymal progenitor cell population as a first step in understanding its role in bladder fibrosis. Using fluorescence activated cell sorting (FACS), we identified a Sca-1^+^/ CD34^+^/ lin^-^ (PECAM^-^: CD45^-^: Ter119^-^) population in the adult murine bladder. These cells were localized to the stromal layer of the adult bladder and appeared by postnatal day 1. Cultured Sca-1^+^/ CD34^+^/ lin^-^ bladder cells self-renewed, formed colonies and spontaneously differentiated into cells expressing smooth muscle genes. These cells differentiated into other mesenchymal lineages (chondrocytes, adipocytes and osteocytes) upon culture in induction medium. Both acute and partial obstruction of the bladder reduced expression of CD34 and changed localization of Sca-1 to the urothelium. Partial obstruction resulted in upregulation of fibrosis genes within the Sca-1^+^/CD34^+^/lin^-^ population. Our data indicate a resident, mesenchymal stem cell population in the bladder that is altered by bladder obstruction. These findings provide new information about the cellular changes in the bladder that may be associated with bladder fibrosis.

## Introduction

Pediatric patients with posterior urethral valves, neurogenic bladder from spina bifida, and other obstructive uropathies are at high risk for kidney injury [1]. In fact, obstructive uropathy is the cause of renal transplantation in up to a quarter of pediatric patients with end stage renal disease [2]. This occurs because after obstructive injury the bladder wall remodels to become either thinner (atonic bladder) or thicker (fibrotic bladder). In either case, urine is not properly voided resulting in retrograde pressure on the ureters and kidney injury. These types of remodeling are reminiscent of other hypertrophic or fibrotic remodeling in injured smooth muscle invested organs. For example, if vascular pressure increases in pulmonary arteries before the typical change in fetal-to-newborn circulation, the vessel walls become abnormally hypertrophied causing pulmonary hypertension [3].

Currently little is understood about which cells proliferate within or migrate to the bladder after obstruction. Studies have demonstrated that bone marrow derived cells are recruited to the mouse bladder after injury [4,5]. However, this is a modest response and it does not appear to lead to robust proliferation of these cells in the bladder. Thus, in cases where bladder injury leads to cell proliferation [6,7], cells intrinsic to the bladder appear to be the primary source of new cell growth after obstruction.

The bladder consists of four layers: a superficial mesothelial layer called the serosa; a smooth muscle layer; a connective tissue layer called the stroma; and an inner layer called the urothelium. Bladder intrinisic progenitors have been identified in the mouse urothelium [6]. Cre-based lineage analysis of these cells after bladder infection revealed that urothelial progenitors proliferated in response to injury but their descendants remained in the urothelium. Urothelial progenitors thus appear committed to the urothelial fates, and do not contribute to the stroma, smooth muscle or serosa after injury.

Resident mesenchymal stem cells (MSCs) have been identified in other muscular organs such as in heart [7], lung [8,9], skeletal muscle [10], and vascular smooth muscle [11–14]. These progenitor cell populations have been shown to play an active role in the deterioration of muscle function in coronary artery vein grafts as well in the restoration of function during muscle remodeling and regeneration [15,16]. Cardiac progenitor cells (CPCs) have been identified in human pediatric patients with congenital heart disease and found to be at higher levels in younger patients. Furthermore, human CPCs were shown to promote a greater degree of wound healing than cardiomyocytes in a cardiac ischemia model [17]. Similar to other muscular organs, the bladder is a plastic organ, demonstrating significant hypertrophic tissue remodeling in response to stimuli such as cystectomy, obstruction or denervation. However, the role of resident MSCs in bladder tissue remodeling has never been described.

Here we set out to determine if MSCs exist in the adult mouse bladder. We focused on cells that express the genes stem cell antigen-1 (Sca-1) and CD34. Co-expression of these genes has been identified as a potential marker for murine progenitor cells in smooth, and skeletal muscle organs. For example, in mouse arterial adventitia, a population of Sca1^+^/CD34^+^ progenitors were identified that spontaneously differentiated into smooth muscle cell in culture [14,18]. Similarly in mouse lung, Sca1^+^/CD34^+^ progenitors were detected that play a role in distal lung homeostasis [8]. Here we show that cells within the stromal layer of the mouse bladder co-express Sca-1 and CD34 and these cells can be isolated by FACS. FACS sorted bladder Sca-1^+^/CD34^+^/lin^-^ cells form colonies in culture and these colonies spontaneously differentiate into cells that express smooth muscle specific genes. We argue that these cells could be intrinsic MSCs that could play a role in bladder remodeling after obstruction.

## Materials and Methods

### Experimental Animals

The Institutional Animal Care and Use Committee of the Stanley Manne Children’s Research Institute approved all animal studies. For some studies we used CD1 mice (Charles River). In other cases bladders were removed from *Rosa* ^*mTmG*^ in a mixed *B129SV/J/C57BL6* background. This Cre reporter strain was used so that the membrane of each cell was labeled with red fluorescence. *Sca-1* ^*egfp*^ (stock # 012643) [19] and *Gli1Cre* ^*ERT2*^ (stock # 007913) [20] mice were obtained from Jackson Laboratories (Bar Harbor, Maine).

### Acute Bladder Outlet Obstruction Surgery (aBOO)

Adult CD1 mice were used in acute obstruction surgeries. A Matrix Medical Inc. Spartan VMC anesthesia unit was used to induce anesthesia. Mice were placed in the induction chamber, which was filled with 3% vaporized isoflurane (Baxter) at a flow rate of 1.5 L/min. Mice were then transferred to the operating table and kept under anesthesia using a rodent anesthesia mask to deliver a continuous flow of isoflurane. To create acute outlet obstruction, a ligature was tied down over a 22G angiocatheter placed in the urethra. Following removal of the catheter, the urethra was fully occluded. For sham surgeries, an incision was made in the abdomen, and then sutured. Post-operatively, mice were placed in a recovery cage that was placed on a heating pad until normal ambulation was observed. Recovery time was typically one hour or less. For analgesic purposes, 500 mg acetaminophen was pulverized and mixed into 250 mL of water and placed in the cage’s water bottle. Mice were subsequently evaluated for pain by observing hunched posturing, decreased ambulation, and/or ruffled fur. If any of these signs were observed, the animal was immediately euthanized. Mice were maintained for 24 hours prior to tissue harvest, at which time they were euthanized by placement in a chamber that was filled with 100% carbon dioxide at a volume replacement rate of 10–30% per minute. Mice were left in the chamber for five minutes. This was followed by cervical dislocation of the animal.

### Partial Bladder Outlet Obstruction Surgery (pBOO)

Adult mice (CD1 or Sca-1^egfp^) were used in partial outlet obstruction surgeries. Anesthesia was performed as above. To create a partial outlet obstruction, a 23G angiocatheter was placed alongside the urethra. A 7.0 Prolene suture was then tied around both the catheter and the urethra. The catheter was then removed, leaving the suture tied around the urethra. Abdomen was then closed with a 5.0 vicryl suture. For sham surgeries, an incision was made in the abdomen and then sutured. Post-operative care was performed as above. For pBOO, mice were allowed to recover for 7 days prior to tissue harvest. Periodically, mice were evaluated for outlet obstruction by voiding stain on paper (VSOP). Euthansia was performed as above.

### Voiding Stain on Paper

Following partial outlet obstruction surgery, mice were evaluated on post-operative day 7 via VSOP[21]. Mice were separated into individual metabolic cages that contained Whatman paper on the floor and kept there for 2 hours. Whatman paper was then imaged using UV light.

### Quantitative PCR

Bladders were removed and placed in ice-cold Trizol reagent (Life Technologies). Samples were either frozen at -80°C or processed immediately. Tissue was thawed and homogenized using either a Polytron PT 1200 Homogenizer (Kinematica AG) or a *BeadBug* (Benchmark Scientific) with 3mm zirconium beads. Nucleic acid was extracted using chloroform, further purified using an RNeasy kit (Qiagen) and precipitated with LiCl. Sorted cells were sorted into RNALater (Ambion) and RNA was extracted via RNeasy kit (Qiagen). Plated cells were washed twice in PBS and then left to incubate in Trizol for 5 minutes at room temperature. A kit geared toward low volume RNA, Direct-zol RNA MiniPrep kit (Zymo Research), was used to extract RNA from plated cells. cDNA was made using high capacity cDNA kit from Applied Biosystems (Invitrogen). qPCR was performed using the Solaris system from Dharmacon Thermo Fisher or TaqMan on an Applied Biosystems StepOne Plus thermocycler. Primers were obtained from Assays-on-Demand (Applied Biosystems) and the primers we used are shown in Table D in S1 File.

### Immunofluorescence of Mouse Bladders

Whole bladders from freshly sacrificed adult and postnatal day 1 (P1) CD1 mice were fixed in 4% formaldehyde (from paraformaldehyde) in phosphate-buffered saline (PBS) overnight at 4°C. They were then transferred sequentially to 20% (wt/v) sucrose in PBS and 30% (wt/v) sucrose in PBS until saturated. The tissue was embedded in OCT (Tissue-Tek) and frozen in a dry ice isopentane slurry. Sections of 10 μm were cut for all experiments. Sections were washed twice in PBS, incubated for 5 minutes at room temperature in PBS + 0.1% Tween (PBT), and blocked for 30 minutes at room temperature with 10% donkey in PBT (or 10% goat serum in PBT, depending on the animal in which the secondary antibody was raised). All primary antibodies were used at 1:100 dilution and left on overnight at 4°C. Slides were then washed 3 times in PBS for 5 minutes. All secondary antibodies were used at a 1:400 dilution and left on for 2 hours at room temperature. Slides were then washed 3X for 5 minutes at room temperature and mounted with Fluorogel (Electron Microscopy Sciences). A complete list of primary and secondary antibodies used can be found in the Tables A and B in S1 File.

### Whole Bladder Digestion to Single Cells

Adult mice were sacrificed and whole bladders were transferred to cold, sterile PBS. Bladders were then minced finely with a sterile razor blade and placed into a dispase II (2.7 mg/mL)/collagenase (1mg/mL) solution at 37°C [22]. Tissue was disrupted ~20 minutes with gentle pipetting until tissue was digested to single cells. Average time of digestions was ~90 minutes. The reaction was stopped using cold FACS buffer (PBS supplemented with 5% fetal bovine serum (FBS) and 10mM HEPES). Cells were filtered through a 70 μm mesh filter, and centrifuged at 150 x g for 5 min at 4°C. The supernatant was removed and the cells were suspended in pure FACS buffer. Cells were then counted using a hemacytometer and volume of FACS buffer was adjusted to yield a cell concentration ranging from 2x10^6^ cells/ml– 10x10^6^ cells/ml for subsequent antibody staining.

### Fluorescence Activated Cell Sorting

FACS of mesenchymal stem cells was based on the work of Houlihan *et al*., 2012 [23]. Cells were stained with antibodies from eBioscience at 1:100 dilution. Cells were stained with conjugated antibodies against Sca-1, CD34, Ter119, CD45 and CD31 for 30 minutes in the dark on ice. Conjugated antibody information can be found in Table C of S1 File. Cells were then washed twice in FACS buffer. Single stain and no stain controls were also prepared for proper compensation. The cells were stained with propidium iodide (Sigma-Aldrich, 1:10 dilution) or Sytox Red (1:250 dilution) 15 minutes prior to the sort to screen out dead cells. Compensation was performed using the compensation setup tool in FACSDiva software (BD Biosciences). Live cells were then sorted for Sca-1^+^/ CD34^+^/lin- using a FACSAria II and FACSDiva software. Post analysis of the sort was performed using Flowjo (Tree Star).

### Crystal Violet Colony Forming Assays

Cells were stained and sorted as mentioned above. Cells were plated at a density of 2000 cells per 10 cm dish and allowed to grow for 2 weeks at 37°C in 5% CO_2_. The medium (α-MEM supplemented with 2mM Glutamax, 10% FBS, and penicillin/streptomycin) was changed every 3 days. After two weeks, cells were washed twice with PBS, and then fixed with 4% formaldehyde in PBS for 10 minutes at room temperature. After 2 washes in PBS, cells were stained with 0.1% (w/v) crystal violet in ethanol for 10 minutes at room temperature. Plates were washed extensively with deionized water and allowed to dry.

### Immunofluorescence of Colony Forming Assays

Sca-1^+^/CD34^+^/Lin^-^ cells were stained and sorted as mentioned above. Cells were plated at a density of 700 cells per well in a 6 well plate lined with cover slips and cultured in α-MEM supplemented with 2mM Glutamax, 10% FBS, and penicillin/streptomycin at 37°C in 5% CO_2_. Medium was changed every 3 days. At various time points (24h, 48h, 4days, and 7d), medium was removed, cover slips were washed twice with PBS, fixed with 4% PFA in PBS for 15 minutes at room temperature, incubated with PBT for 5 minutes at room temperature, and blocked with 10% donkey (or goat) serum in PBT for 30 minutes at room temperature.

### EdU Assay

Cells were prepared as mentioned above and 5-ethynyl-2’-deoxyuridine (EdU) was added to the medium at a concentration of 1 μm. Medium was changed every 3d. EdU stain was visualized using Click-iT EdU Alexa-Fluor 647 Imaging Kit (Molecular Probes).

### Sca-1^+^/CD34^+^/lin^-^ Tissue Culture for qPCR Analysis

Sca-1^+^/CD34^+^/lin^-^ cells were stained and sorted as mentioned above. Cells were plated at a density of 10,000 cells per well in 48 well tissue culture plates (BD Falcon) and cultured in α-MEM supplemented with 2mM Glutamax, 10% FBS, and penicillin/streptomycin at 37°C in 5% CO_2_. This medium was considered to be non-induction medium. Medium was changed every 3 days. At 7 days, cells were processed for RNA as mentioned above in the quantitative PCR section.

### Differentiation Assays

Cells were stained, sorted by FACS and cultured under specific conditions for adipogenic, chondrogenic, or osteogenic differentiation (Lonza). Note: In addition to TGF-β3 in the chondrogenic assay, cells were grown in the presence of BMP-6 (500 ng/mL, Peprotech).

### Oil Red O Assay

Cells were fixed for 30 min. at room temperature in 10% formalin, washed once with milliQ water, incubated with 60% isopropanol for 3 minutes and stained with Oil Red O (Sigma): milliQ Q water (3:2) for 5 minutes. Wells were washed twice with deionized water and coverslips mounted with aqueous mounting medium.

### Alizarin Red Stain

Cells were washed in DPBS and then fixed in 10% formaldehyde (v/v). Cells were washed twice with DPBS and incubated in 2% alizarin red stain for 20 minutes. Cells were washed four times with distilled water and imaged on a Leica inverted microscope.

### Endogenous Alkaline Phosphatase Stain

Cells were fixed in 4% PFA for 30 seconds and then washed 2X with PBS. 200 μl of Naphothol AS-MX (Sigma) in dimethyl formamide was added to 10mL of 0.1M Tris-HCl and vortexed. 10 mg of Fast blue BB (Sigma) was then added to the solution and vigorously vortexed. The solution was subsequently syringe filtered using a 0.2 μm filter. The solution was placed in the wells to stain the cells for 1 hour at room temperature in the dark. Cells were then washed 2X with PBS.

### Toluidine Blue Stain

Chondrogenic pellets were embedded in paraffin blocks and 6 μm sections were cut. Sections were deparaffinized and hydrated with distilled water. Sections were stained in Toluidine Blue O (Sigma) solution at a concentration of 1 mg/mL in water for 30 minutes at room temperature. Sections were then dehydrated once with 95% ethanol for 5 minutes and then 2X for 5 minutes each with 100% ethanol. Sections were then washed 3X for 10 minutes each with xylene and mounted with a coverslip.

### Statistical Analyses

All statistical analyses were done using GraphPad Prism version 5 for Mac OSX, (GraphPad Software, La Jolla, CA). For univariate analysis we employed a Student’s T-Test. For multivariate analysis we tested for significance using one-way ANOVA and employed Tukey’s post-hoc test.

## Results

### Bladder digests contain a population of Sca-1^+^/CD34^+^/lin^-^ MSCs

**Fig 1:**
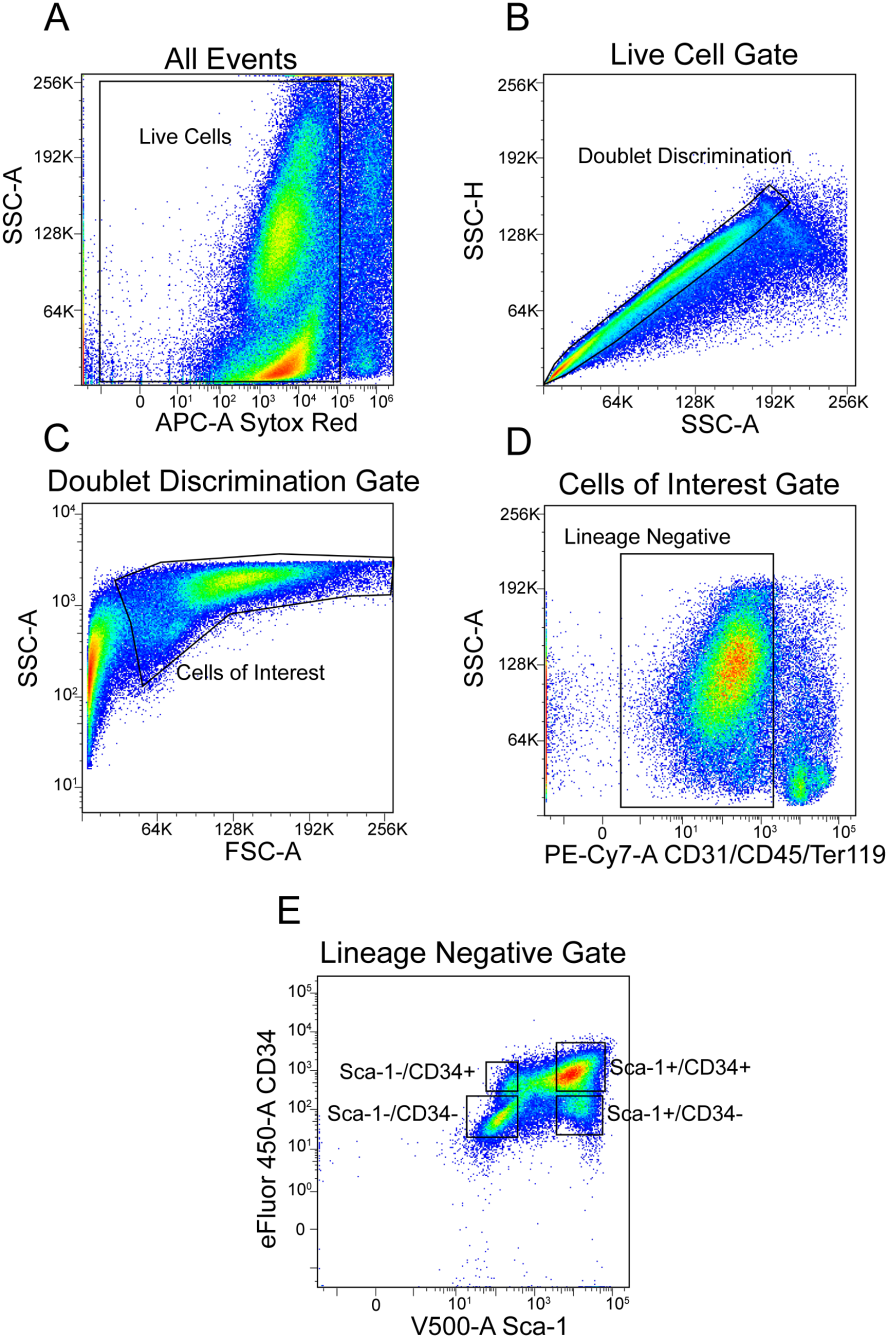
Flow cytometric analysis of adult mouse bladder cells. (A) Density dot plot for bladder cell suspensions sorted based on Sytox red uptake and side-scatter. (B) Gating to exclude doublets. (C) Gating for side scatter versus forward scatter. (D) Gating for side scatter versus lineage (lin) staining (FITC CD31, CD45 and TER-119). (E) Gating of lin^-^ cells for Sca-1 and CD34 expression.

Cell suspensions were made from dispase II, collagenase digests of whole CD1 mouse bladders and analyzed by FACS (Table 1). Unstained (S1 Fig) and single stain controls (S1 Fig) were used for compensation and gating. Propidium iodide or Sytox red uptake was used to gate living cells from dead cells (Fig 1A). In order to target single cells, side scatter height versus side scatter area was used to screen out doublets (Fig 1B) and living cells were further limited by gating based on side scatter area versus forward scatter area (Fig 1C). Bivariate FACS analysis indicated that approximately 25% of the gated cells expressed CD31, CD45 or TER-119 (Table 1). These cells were considered lineage positive (lin^+^) and were gated out of further analysis (Fig 1D). Of the remaining lin^-^ cells, 48.02% were Sca-1^+^ and 51.98% were Sca-1^-^. When cells were gated by virtue of their coexpression of Sca-1 and CD34, we determined that of the total living bladder cells we sorted, approximately 14% co-expressed Sca-1 and CD34, while not expressing the lineage markers CD31, CD45 or TER-119 (Table 1 and Fig 1E). However, this did not represent the entire population of Sca-1^+^ cells. In fact, within the entire Sca-1^+^ population, about 51% co-expressed CD34, with 49% CD34 negative. Looking only at the population that expressed CD34, approximately 84% were Sca-1^+^ and 13% were Sca-1^-^ (3% were not gated). Thus, most CD34^+^ cells co-expressed Sca-1, whereas approximately half of the Sca-1^+^ cells did not co-express CD34.

**Table 1:** Summary of flow cytometric analysis.

*Gate profile*	*Percent living cells*
*lin+*	*25*.*44 ± 3*.*14%*
*Sca-1* ^*+*^ */CD34* ^*+*^ */lin* ^*-*^	*14*.*03% ± 0*.*61%*
*Sca-1* ^*+*^ */CD34* ^*-*^ */lin* ^*-*^	*7*.*64% ± 0*.*80%*
*Sca-1* ^*-*^ */CD34* ^*-*^ */lin* ^*-*^	*25*.*18% ± 2*.*12%*
*Sca-1* ^*-*^ */CD34* ^*+*^ */lin* ^*-*^	*2*.*59% ± 0*.*32%*

Percentages were averaged from 10 different sorting experiments, and represent percentage of total live cells (all Sytox red or propidium iodide negative cells). ± % is standard error of the mean.

**Fig 2:**
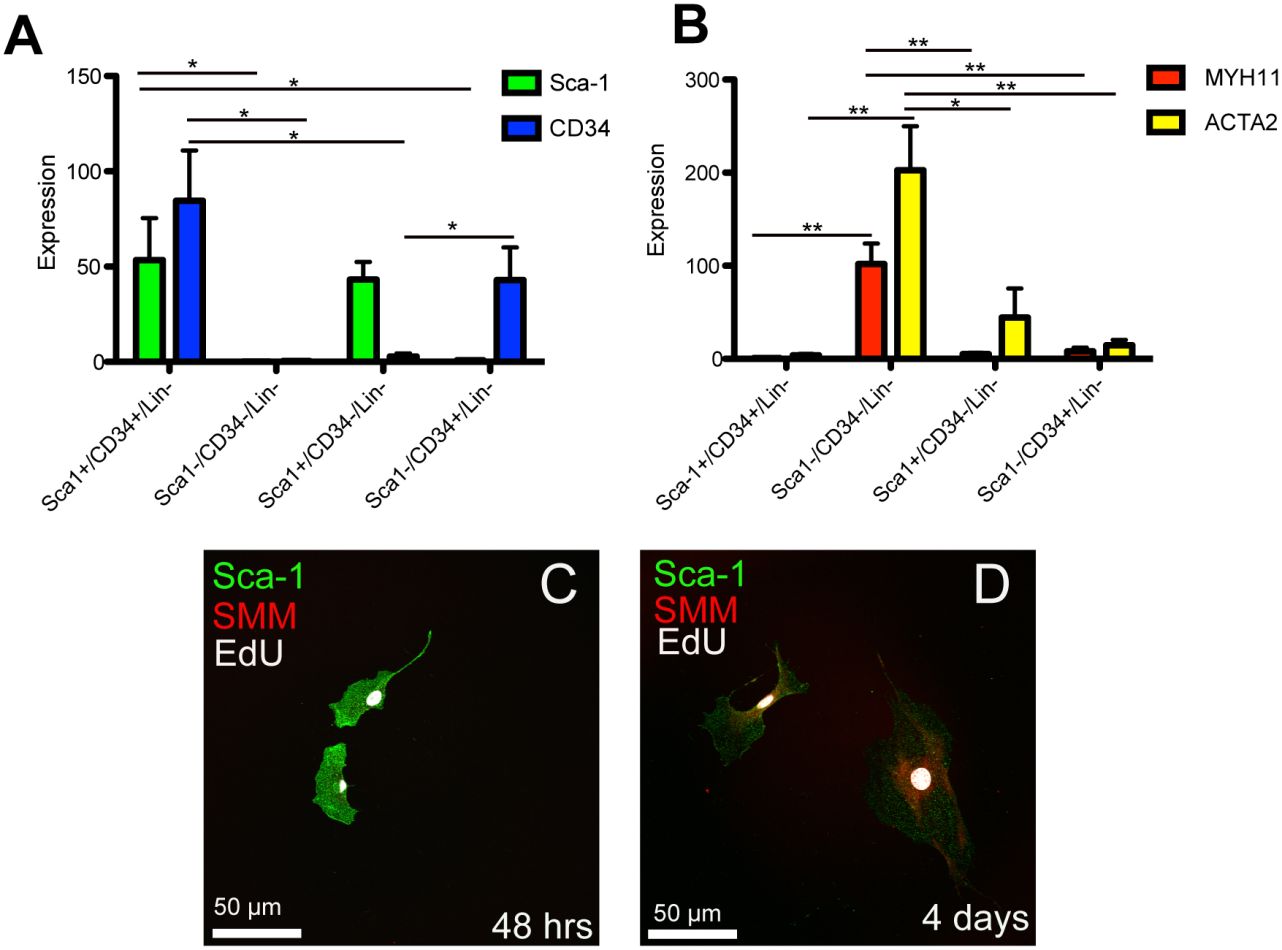
Analysis of gene and protein expression in FACS sorted bladder cells. (A) qPCR analysis of Sca-1 and CD34 mRNA expression levels in lin^-^ cells that were FACS sorted based on expression of Sca-1 and CD34. Expression level is normalized to Sca-1^-^/CD34^-^/lin^-^ Sca-1 expression levels. (B) qPCR analysis of smooth muscle myosin (SMM) and smooth muscle alpha **α** actin (ACTA2) of lin^-^ cells that were FACS sorted based on Sca-1 and CD34 expression levels. Expression level is normalized to Sca-1^+^/CD34^+^/lin^-^ SMM expression levels. (A, B) Asterisks represent significance values of P < 0.05 * and P < 0.01 ** after 1 Way ANOVA. Graphs represent expression averages from 4 separate sorts with 3–4 CD1 mice pooled per sort. (C, D) Confocal micrographs of Sca-1^+^/CD34^+^/lin^-^ sorted cells cultured on a glass coverslips after incubation with EdU for the first 24h (D) Two cells that are Sca-1^+^ (green), EdU^+^ (white) but SMM^-^ (red) at 48h in culture in α-MEM media. (E) Two cells that are Sca-1^+^ (green), EdU^+^ (white) and SMM^+^ (red) at 4d in culture in α-MEM media.

To investigate the identity of the four populations, we FACS sorted cells from adult CD1 bladders and immediately isolated RNA. RNA samples were examined for expression of several genes. Here we determined that Sca-1^+^/CD34^+^/lin^-^ cells expressed Sca-1 and CD34 transcripts (Fig 2A), but did not express smooth muscle **α** actin (*ACTA2*) or smooth muscle myosin (*Myh11*) (Fig 2B). Sca-1^-^/CD34^-^/lin^-^ cells demonstrated the highest levels of expression of *ACTA2* and *Myh11* (Fig 2B), suggesting that many of these cells are smooth muscle cells. This population also expressed relatively high levels of the epithelial marker keratin 5 (*KRT5*), suggesting that this population may contain serosal and urothelial cells (S2 Fig). Sca-1^+^/CD34^-^/lin^-^ and Sca-1^-^/CD34^+^/lin^-^ demonstrated expression of genes that suggest that these populations are heterogeneous. For example, Sca-1^+^/CD34^-^/lin^-^ expressed the highest levels of *Col1* **α** *1* (S2 Fig), which is expressed in connective tissue cells. *ACTA2* was also expressed at low levels in this population suggesting that some of these cells are smooth muscle cells. This population did not express high levels of *Myh11*. The Sca-1^-^/CD34^+^/lin^-^ population expressed relatively low levels of *Col1* **α** *1*, but expressed relatively high levels of *KRT5*, suggesting that some of these cells may be serosal or urothelial cells (S2 Fig). Additionally, Sca-1^-^/CD34^+^/lin^-^ cells did express some *ACTA2*, although at low levels (Fig 2B). This suggests that some of these cells are also smooth muscle cells.

Since Sca-1^+^/CD34^+^/lin^-^ cells uniquely expressed Sca-1 and CD34 and did not express genes associated with differentiated cell types, we used FACS to analyze their ability to form colonies in tissue culture. We plated Sca-1^+^/CD34^+^/lin^-^ cells at low density on plastic tissue culture plates. Total live cells were sorted and cultured under the same conditions as controls. Here we found that Sca-1^+^/CD34^+^/lin^-^ cells robustly formed colonies after two-weeks while control cells did so sporadically (S2 Fig). To investigate if Sca-1^+^/CD34^+^/lin^-^ cells were self-renewing we added the nucleotide analog EdU to culture medium for 24-hours. Cells were grown for 48h or 4d in EdU-free medium before fixation and staining for EdU incorporation and Sca-1. At 48h we found Sca-1 cells that had incorporated EdU indicating that sorted Sca-1^+^/CD34^+^/lin^-^ cells passed through S-phase and continued to express Sca-1 (Fig 2C). At 4d we found cells expressing Sca-1 and smooth muscle myosin (SMM) proteins that had incorporated EdU (Fig 2D). This finding demonstrated that Sca-1^+^/CD34^+^/lin^-^ cells self-renewed during the first 4d of culture.

**Fig 3:**
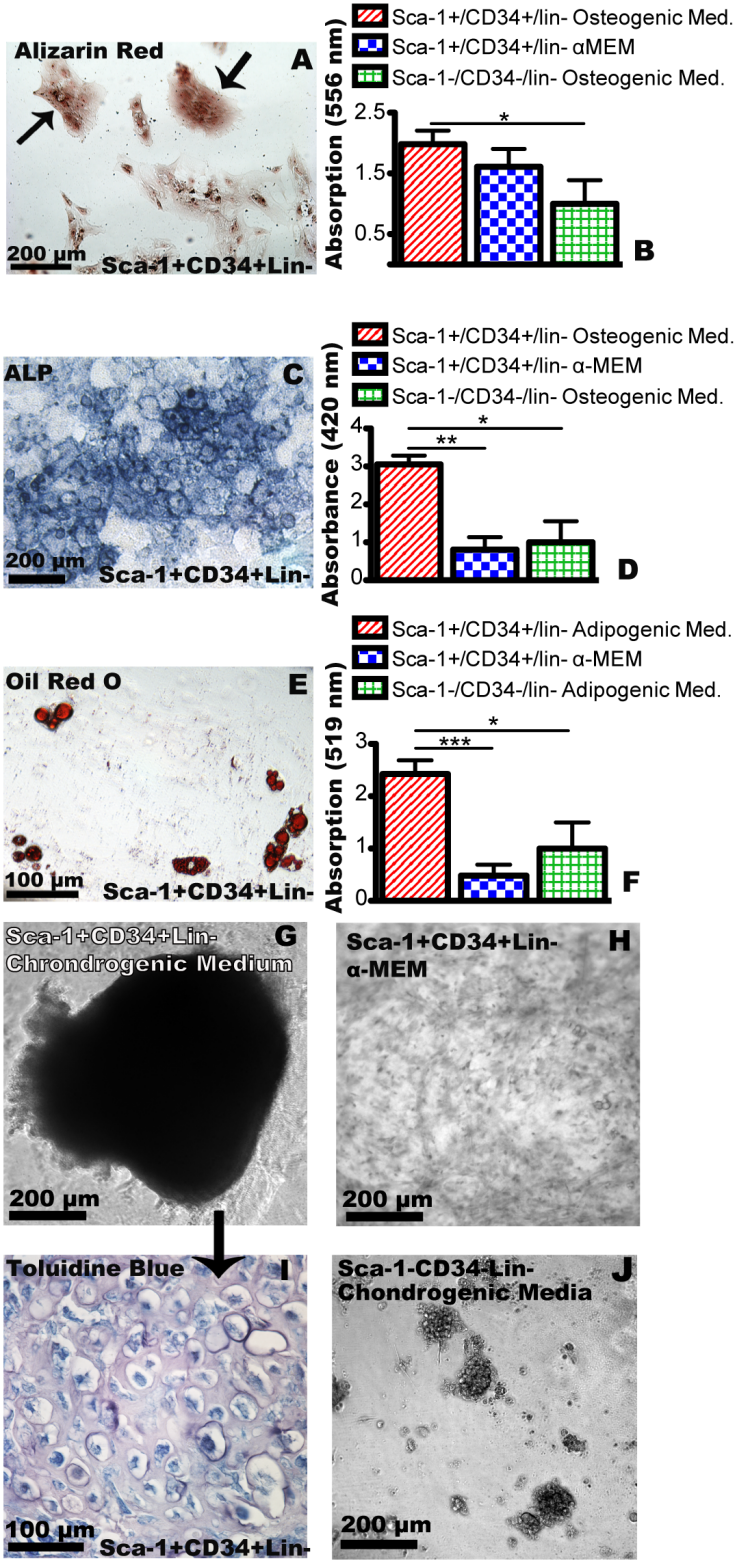
*In vitro* differentiation of mouse bladder mesenchymal stem cells. (A) Sca-1^+^/CD34^+^/lin^-^ cells grown in osteogenic medium, two weeks post-sort, stained with alizarin red for calcium deposition. (B) Quantification of alizarin red by absorbance at 556nm. Red hatched bar represents Sca-1^+^/CD34^+^/lin^-^ cells grown in osteogenic induction medium, blue hatched bars represent Sca-1^+^/CD34^+^/lin^-^ grown in non-induction medium (α-MEM) and green hatched bars represent Sca-1^-^/CD34^-^/lin^-^ cells grown in osteogenic induction medium. (C) Sca-1^+^/CD34^+^/lin^-^ cells grown in osteogenic induction medium two weeks post-sort, stained for endogenous alkaline phosphatase. (D) Quantification of alkaline phosphatase staining in cultures by absorbance at 420nm. (E) Sca-1^+^/CD34^+^/lin^-^ cells grown in adipogenic medium, two weeks post-sort, stained with Oil Red O. Lipid droplets are stained red. (F) Quantification of Oil Red O stain by absorbance at 519nm. (G) Sca-1^+^/CD34^+^/lin^-^ cells grown in chondrogenic induction medium formed pellets two weeks post sorting whereas Sca-1^+^/CD34^+^/lin^-^ cells grown in α-MEM and Sca-1^-^/CD34^-^/lin^-^ cells grown in osteogenic induction medium did not form pellets (H, J). (I) Cross section of chondrogenic pellet such as in (G) stained with toluidine blue. Asterisks in (B), (D) and (F) represent significance values of P < 0.05 *, P < 0.01 ** and P < 0.001 *** after Student’s T-Tests. Scale bars are as shown.

While the exact sorting profile of MSCs is still a topic debate, a hallmark of MSCs is their ability to form colonies and adhere to tissue culture plastic [24]. Because Sca-1^+^/CD34^+^/lin^-^ bladder cells both adhered to tissue culture plastic and formed colonies, we next investigated their ability differentiate into various mesenchymal lineages (Fig 3). Here we found that when bladder derived Sca-1^+^/CD34^+^/lin^-^ cells were placed in induction media, they formed bone (Fig 3A, 3B, 3C and 3D), adipose (Fig 3E and 3F) and cartilage (Fig 3G and 3I) cells. Brightfield images (S2 Fig; Fig 3H and 3J) coupled with staining (Fig 3B, 3D and 3F) suggest that Sca-1^+^/CD34^+^lin^-^ cells cultured in α-MEM alone or Sca-1^-^/CD34^-^/lin^-^ cultured in induction medium did not differentiate into these cells types. Thus, EdU incorporation into cells that continued to express Sca-1 and differentiation into bone, cartilage and fat in specialized medium supported the idea that Sca-1^+^/CD34^+^/lin^-^ bladder cells could represent a resident MSC population.

**Fig 4:**
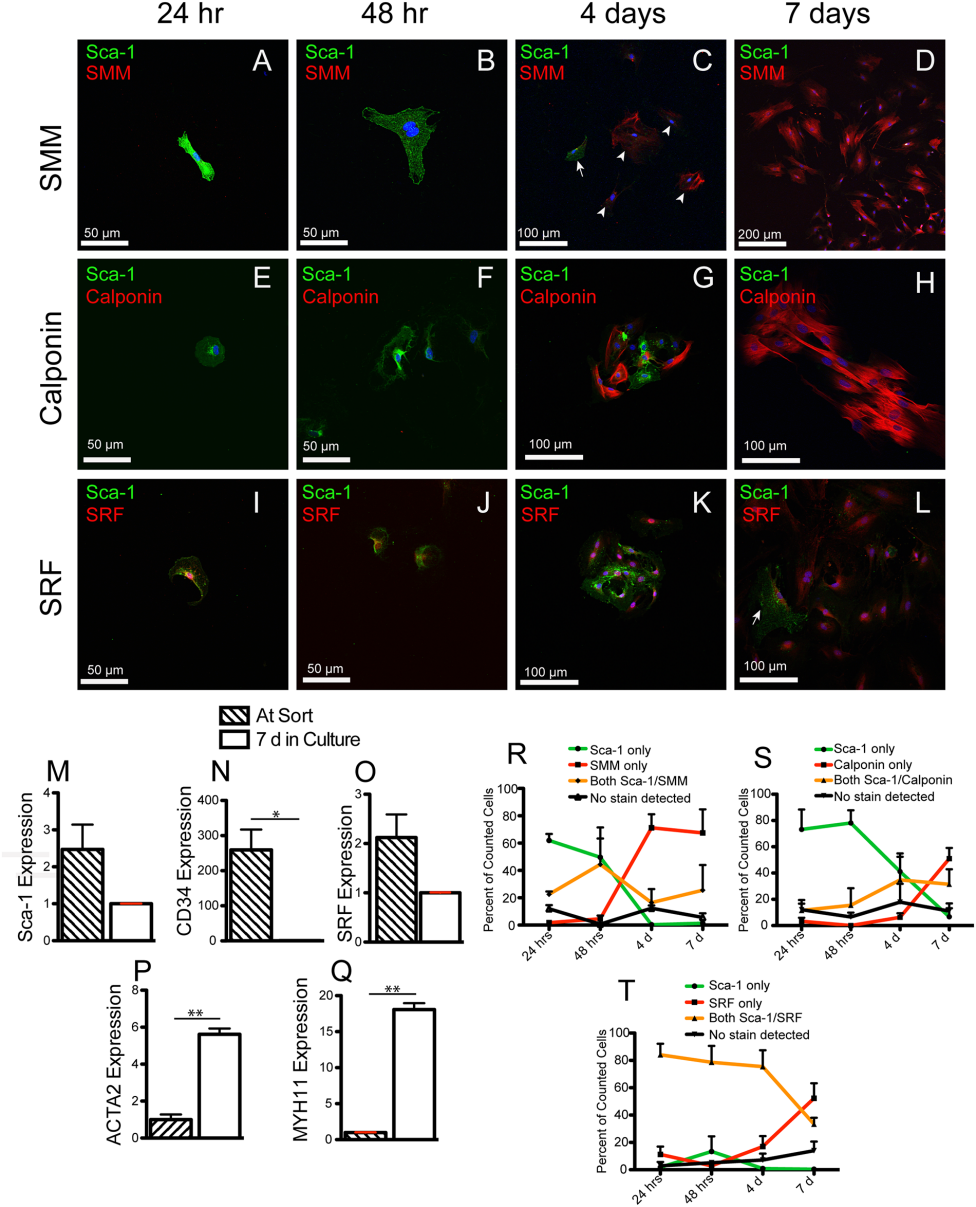
Colony forming assays with Sca-1^+^/CD34^+^/lin^-^ cells demonstrate that individual cells form colonies and differentiate into cells expressing smooth muscle genes. (A-D) Confocal micrographs of Sca-1^+^/CD34^+^/lin^-^ sorted cells cultured on glass coverslips and stained with antibodies to Sca-1 (green) and SMM (red) at varying time points up to 7 days. Arrow in (C) shows a single Sca-1^+^ cell next to a group of SMM^+^ cells (arrowheads) at 4d of culture. (E-H) Confocal micrographs of Sca-1^+^/CD34^+^/lin^-^ sorted cells cultured on glass coverslips in α-MEM and stained with antibodies to Sca-1 (green) and calponin (red) at varying time points up to 7 days. (I-L) Confocal micrographs of Sca-1^+^/CD34^+^/lin^-^ sorted cells cultured on glass coverslips in α-MEM and stained with antibodies to Sca-1 (green) and SRF (red) at varying time points. Arrow (L) shows a single cell co-expressing SRF and Sca-1 next to a group of SRF^+^, Sca-1^-^ cells stained in red. (M-Q) qPCR analysis of Sca-1^+^/CD34^+^/lin^-^ expression of 5 genes at the time of sort versus after 7 days in α-MEM culture. *Sca-1*, *CD34*, and *SRF* expression levels are normalized to expression levels after 7 d in culture. *Myh11* and *ACTA2* are normalized to expression levels at the time of sort. Analysis represents two technical replicates of two separate sorts. Each sort consisted of 5 or 6-pooled CD1 mouse bladder cells. Asterisks (N, P, Q) represent significance values of P < 0.05 * and P < 0.01 ** after Student’s T-test. (R, S) Quantification of spontaneous *in vitro* differentiation Sca-1^+^/CD34^+^/lin^-^ cells into SMM and calponin expressing cells. (T) Quantification of *in vitro* expression of SRF and Sca-1 in Sca-1^+^/CD34^+^/lin^-^ cells. (R, S, T) Cells from three independent sorting experiments were fixed at 24h, 48h, 4d and 7d. Coverslips were stained for Sca-1 and either SMM, calponin or SRF. Green lines represent cells stained only with Sca-1. Gold lines represent cells stained with Sca-1 and SMM, Sca-1 and calponin or Sca-1 and SRF. Red lines represent cells stained with SMM, calponin or SRF but not Sca-1. Black lines represent cells that did not stain at all. Error bars (R-T) represent standard error of the mean.

We cultured small numbers of Sca-1^+^/CD34^+^/lin^-^ cells in non-induction medium and periodically fixed them for immunofluorescence analysis (Fig 4). We used antibodies against mature smooth muscle cytoskeletal proteins (SMM and calponin) and SRF, a transcription factor expressed early in smooth muscle development [25]. After 24h and 48h, the majority of sorted cells expressed Sca-1 and SRF, but not SMM or calponin, (Fig 4A, 4B, 4E, 4F, 4I and 4J). After 4d, Sca-1^+^/SMM^+^, Sca-1^-^/SMM^+^, Sca-1^+^/calponin^+^ and Sca-1^-^/calponin^+^ cells were observed in colonies, suggesting that the cells that were initially sorted were dividing and daughters were becoming cells that expressed SMM and calponin (Fig 4C, 4G, 4R and 4S). After 7d, while some Sca-1^+^ cells remained (Fig 4L, arrow), most cells in colonies were positive for SMM, calponin and SRF (Fig 4D, 4H and 4L). qPCR analysis of Sca-1^+^/CD34^+^/lin^-^ cells at the time of sort versus after 7 days in α-MEM culture was consistent with our findings in immunofluorescence analysis (Fig 4M–4Q). After 7d in culture, *Sca-1* expression decreased by approximately 1.2-fold and *CD34* expression decreased by over 250-fold (Fig 4M and 4N). A 5-fold increase in *ACTA2* and an 18-fold increase SMM (*Myh11*) expression was observed at 7d in culture. (Fig 4P and 4Q). *SRF* levels decreased by about half after 7d in culture (Fig 4O and 4T). These observations, combined with our immunofluorescence analysis supported the hypothesis that Sca-1^+^/CD34^+^/lin^-^ cells acquire properties of smooth muscle cells when cultured in non-induction medium.

### Sca-1^+^ and CD34^+^ expression is primarily localized in stromal layer cells of the adult mouse bladder

**Fig 5:**
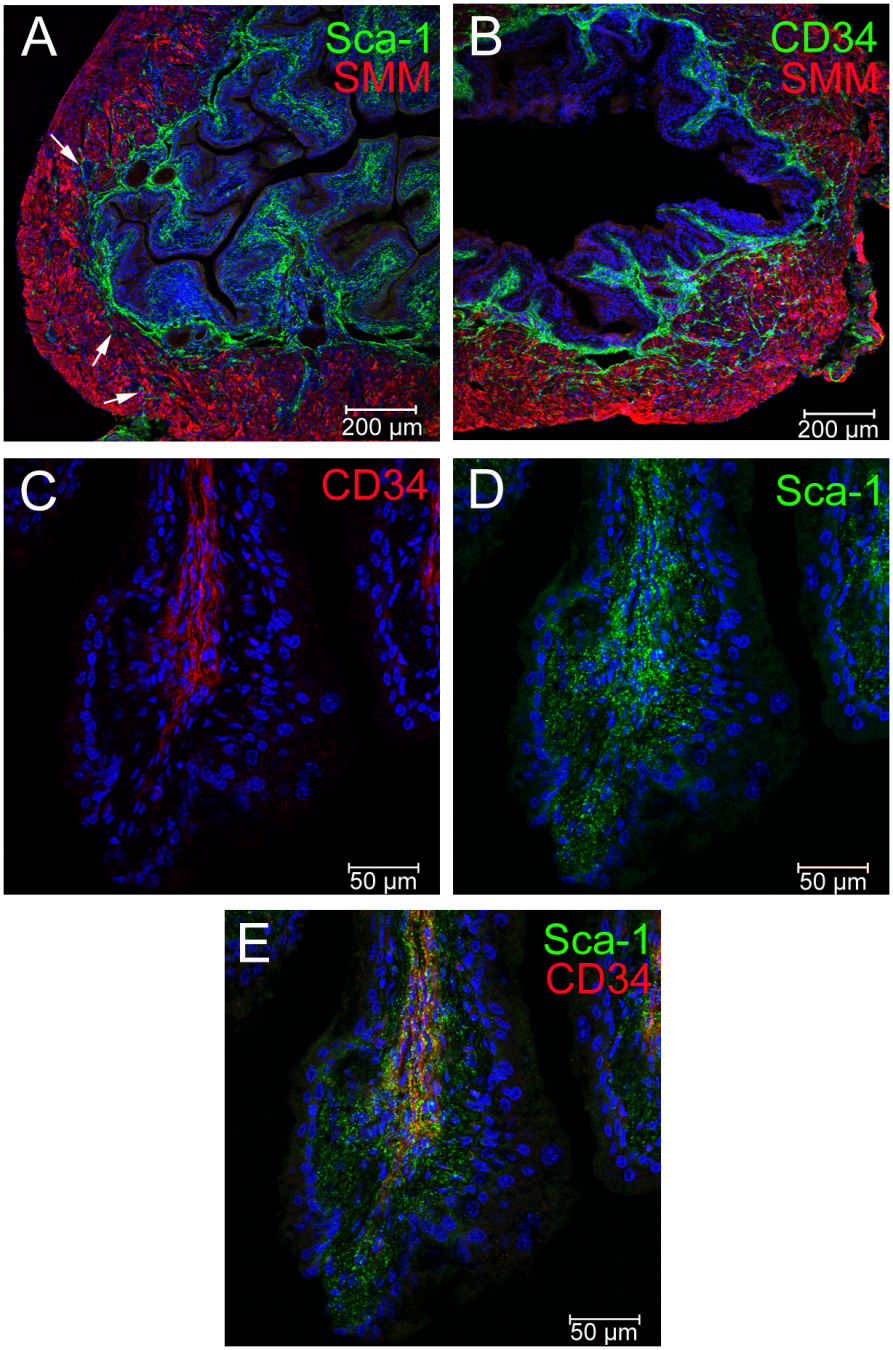
Co-expression of Sca-1 and CD34 in the stromal layer of the normal CD1 adult bladder. Fluorescent confocal images of bladder sections demonstrating regions of the adult mouse bladder that react with antisera to Sca-1 (A, D, E, green) and CD34 (B, green, C, E, red). Sections were stained with anti-SMM (A, B, red) to label the bladder wall. Co-labeling with anti-Sca-1 and anti-CD34 (D-F) demonstrates areas of overlapping expression in the stroma. Scale bars are as shown.

To determine the localization of Sca-1^+^/CD34^+^/lin^-^ cells in the adult bladder we examined sectioned mouse bladders from adult (6–8 week old) CD1 mice for cells expressing Sca-1 and CD34 (Fig 5). We found that there was robust expression of Sca-1 in mesenchymal cells of the stromal layer lying in between the urothelial and smooth muscle layers of the bladder. This expression pattern sporadically overlapped with the basal epithelial layer of the urothelium, an area that was previously defined as containing epithelial stem cells of the urothelium [6]. Cells reacting with the Sca-1 antibody extended from the stromal layer into the smooth muscle layer of the bladder (Fig 5A, arrows). CD34 expression was also prominent in the stromal layer (Fig 5B). This expression was more clearly demarcated from the urothelium as there was no detectable staining near the basal urothelium (Fig 5B and 5C). When Sca-1 and CD34 were co-localized (Fig 5E) it was clear that CD34 expressing cells are positioned more centrally within the stromal mesenchyme, whereas Sca-1 is found throughout the stroma and in isolated cells of the urothelium. This observation was consistent with our FACS data in which we had observed that approximately 51% of Sca-1^+^ cells expressed CD34 and that most CD34^+^ cells express Sca-1.

**Fig 6:**
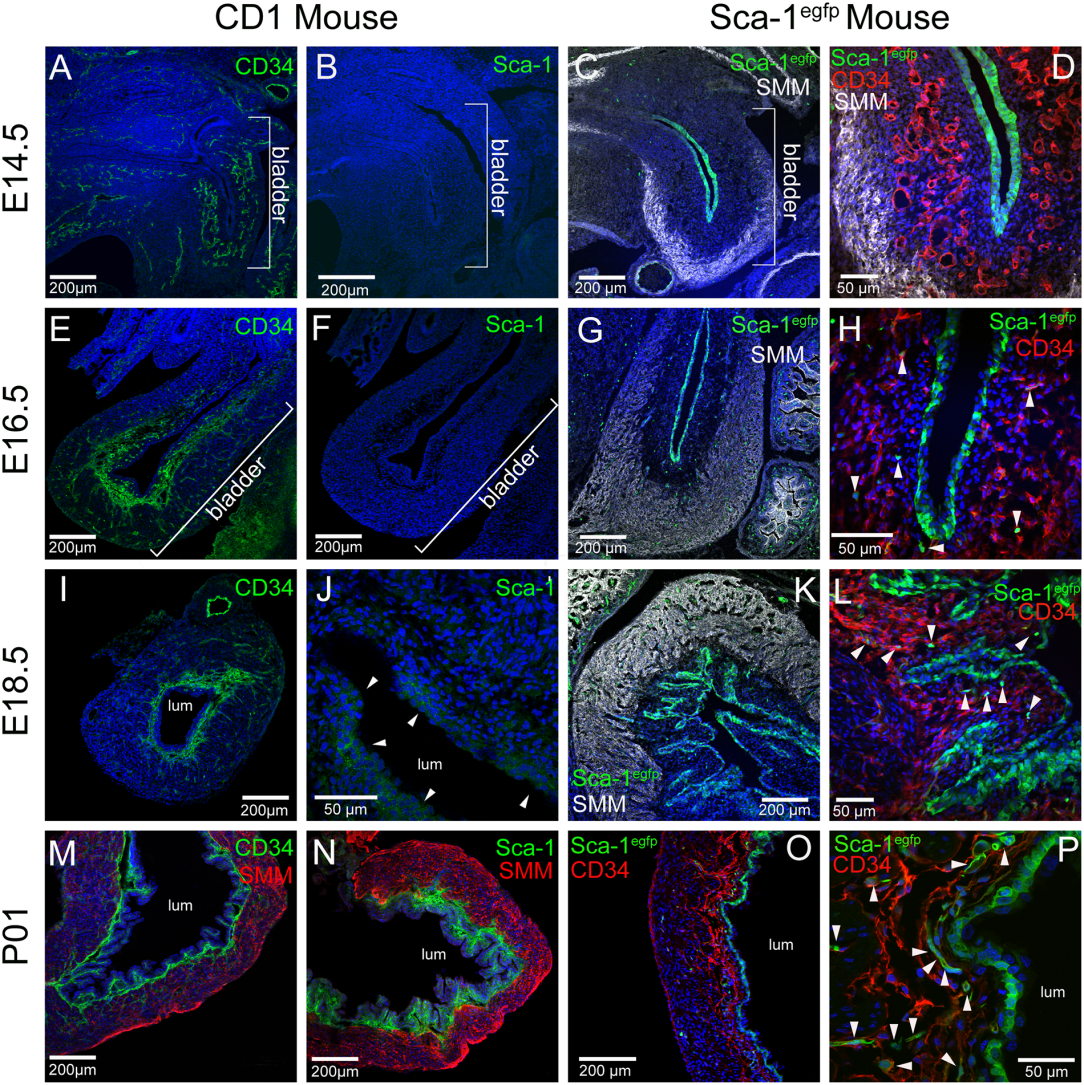
Appearance of a Sca-1^+^/CD34^+^ cell population in the bladder stroma by postnatal day -1. Embryonic CD1 mouse bladders were stained at various time points in development for CD34 (A, E, I, M) and Sca-1 (B, F, J, N). Embryonic *Sca-1* ^*egfp*^ mouse bladders were stained at various time points for smooth muscle myosin (C, D, G, K) and CD34 (D, H, L, O, P). EGFP (green) was detected endogenously. The age of the bladders is shown to the left of the panels. In some cases the lumen of the bladder is indicated (lum). Arrowheads in (J) point to cells that have reacted with anti-Sca-1 at this stage. Arrowheads (D, H, L, P) point to EGFP expressing cells co-localized to the CD34 expressing cells of the developing stroma. Scale bars are as shown.

To investigate if stromal Sca-1^+^/CD34^+^ cells are present during fetal development we stained sections from CD1 embryonic bladders (Fig 6). As early as E14.5, CD34 expression was localized throughout the bladder wall and in portions of the differentiating smooth muscle layer of the bladder (Fig 6A). CD34 was observed throughout the E16.5 bladder, more dispersed through the bladder wall and in what appears to be a condensing stromal layer (Fig 6E). At E18.5, CD34 expressing cells were numerous in what is now clearly the stromal layer of the bladder (Fig 6I). The distribution of these cells narrowed over time to correspond to a compact stromal compartment at P01 (Fig 6M). Sca-1 protein, on the other hand, was not detected in embryonic bladders until E18.5 (Fig 6B, 6F, 6J and 6N) while it was detected in other tissues of the embryo (data not shown). At E18.5, the Sca-1 antibody reacted with a small number of urothelial cells (Fig 6J, arrowheads). In P01 sections, Sca-1 localization overlapped the stromal compartment of the bladder (Fig 6N). It was not until P01 that we observed robust co-localization of CD34 and Sca-1 protein in the stromal layer (Fig 6M and 6N). Thus, CD34 expression precedes Sca-1 expression in the stromal mesenchyme *in utero*, but both are established by the first day of life.

To investigate whether the Sca-1 promoter was active early in bladder development, we examined tissue from *Sca-1* ^*egfp*^ mice [19]. Surprisingly, the Sca-1 gene demonstrated activity at these embryonic stages in the urothelium (Fig 6C, 6G, 6K and 6O). No EGFP was detected in areas where we observed CD34 localization at E14.5 (Fig 6D). However, at E16.5, E18.5 and P01, EGFP was detected in the developing stroma in increasing amounts (arrowheads, Fig 6H, 6L and 6P, arrowheads) and partially overlapped with CD34 expression. In 4-6wk old mice, EGFP was only observed in the stroma and in isolated cells of the detrusor muscle, but not in the urothelium (S3 Fig). This demonstrated that there is a shift in gene expression of Sca-1 from the urothelium in embryos to the stroma and detrusor in adults. The inability to detect Sca-1 protein at earlier stages may reflect a delay in accumulation of the Sca-1 antigenic determinant in the urothelium.

### A subset of stromal Sca-1^+^/CD34^+^/lin^-^ cells respond to Shh in the adult bladder

**Fig 7:**
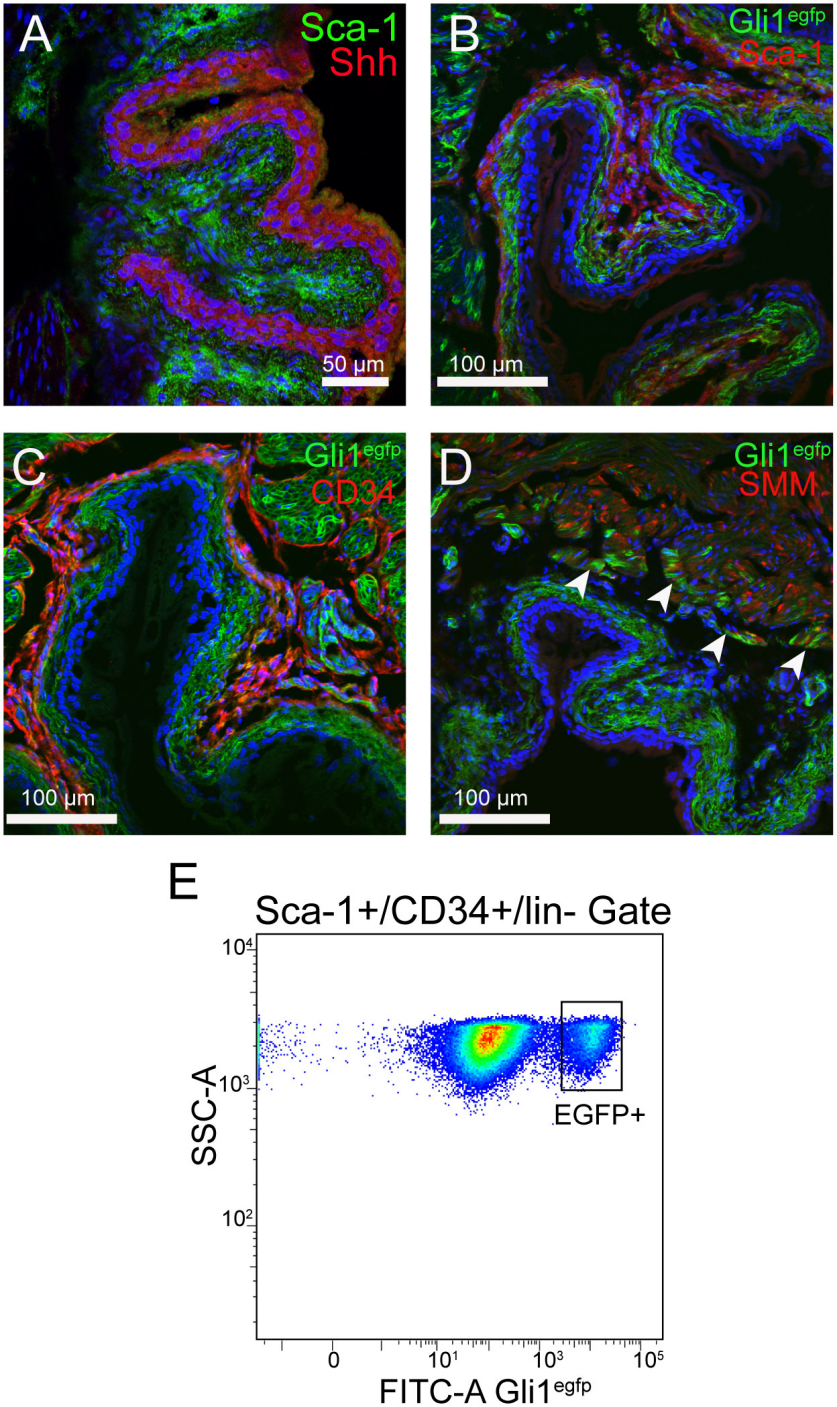
A subpopulation of Sca-1+/CD34+/lin- cells responds to sonic hedgehog (Shh) signaling. (A) Fluorescent confocal image of CD1 adult bladder stained with Sca-1 (green) in the stroma and Shh (red) in the urothelium. (B) *Gli-1* ^*CreERT2/+*^ *; Rosa* ^*mTmG+*^ EGFP^+^ cells (green) shown in the bladder stroma partially overlap with Sca-1^+^ cells (red). (C) EGFP^+^ cells (green) in the bladder stroma partially overlap with CD34^+^ cells (red). (D) EGFP^+^ cells (green) in the smooth muscle layer (red) are localized to a region of the detrusor layer near the stromal layer. (arrowheads). (E) 10% of Sca-1^+^/CD34^+^/lin^-^ cells from *Gli-1* ^*CreERT2/+*^ *; Rosa* ^*mTmG+*^ mouse bladders gated for SSC-A and FITC-A Gli1^egfp^ express EGFP. FACS analysis is from two separate sorts each containing bladder digests from two *Gli-1* ^*CreERT2/+*^ *; Rosa* ^*mTmG+*^ mice.

The urothelium is a source of sonic hedgehog (Shh) signaling in the bladder [26]. It is also known that other Sca-1^+^/CD34^+^/lin^-^ mesenchymal stem cell populations reside in Shh signaling domains [14]. We investigated if the stroma of the adult mouse bladder contained cells responding to Shh signals. We stained sections of adult CD1 bladders with an antiserum to Shh and observed that Shh was largely expressed by the urothelium (Fig 7A). We next used a tamoxifen inducible *Cre* gene that is driven by the *Gli1* promoter, which is activated in response to *Shh* signaling [20]. We generated mice carrying *Gli1Cre* ^*ERT2*^ and the Cre reporter *Rosa* ^*mTmG*^. To obtain a “snapshot” of cells responding to Shh we injected one dose of tamoxifen (4mg) three days before bladders were harvested for analysis by fluorescence microscopy. Here we observed two main sites of Cre labeled cells. First we observed cells in the stroma, particularly in the layers directly adjacent to the urothelium (Fig 7B and 7C). Partial overlap between Gli1 driven EGFP, Sca-1 and CD34 was also observed (Fig 7B and 7C). This was consistent with the hypothesis that some Sca-1^+^/CD34^+^/lin^-^ mesenchymal stem cells are responding to Shh. We also observed a significant number of cells in the detrusor muscle, primarily in cells closest to the stromal compartment (Fig 7D, arrowheads). Using the same method of tamoxifen administration, we labeled cells in adult bladders and analyzed the cells by FACS. Here we observed that approximately 10% of the Sca-1^+^/CD34^+^/lin^-^ population was labeled by recombination of *Rosa* ^*mTmG*^ (Fig 7E). Together, these observations support a model in which bladder Sca-1^+^/CD34^+^/lin^-^ MSCs exist in a Shh signaling domain, but are not all directly responding to Shh.

### Expression of Sca-1 and CD34 is altered after bladder outlet obstruction

**Fig 8:**
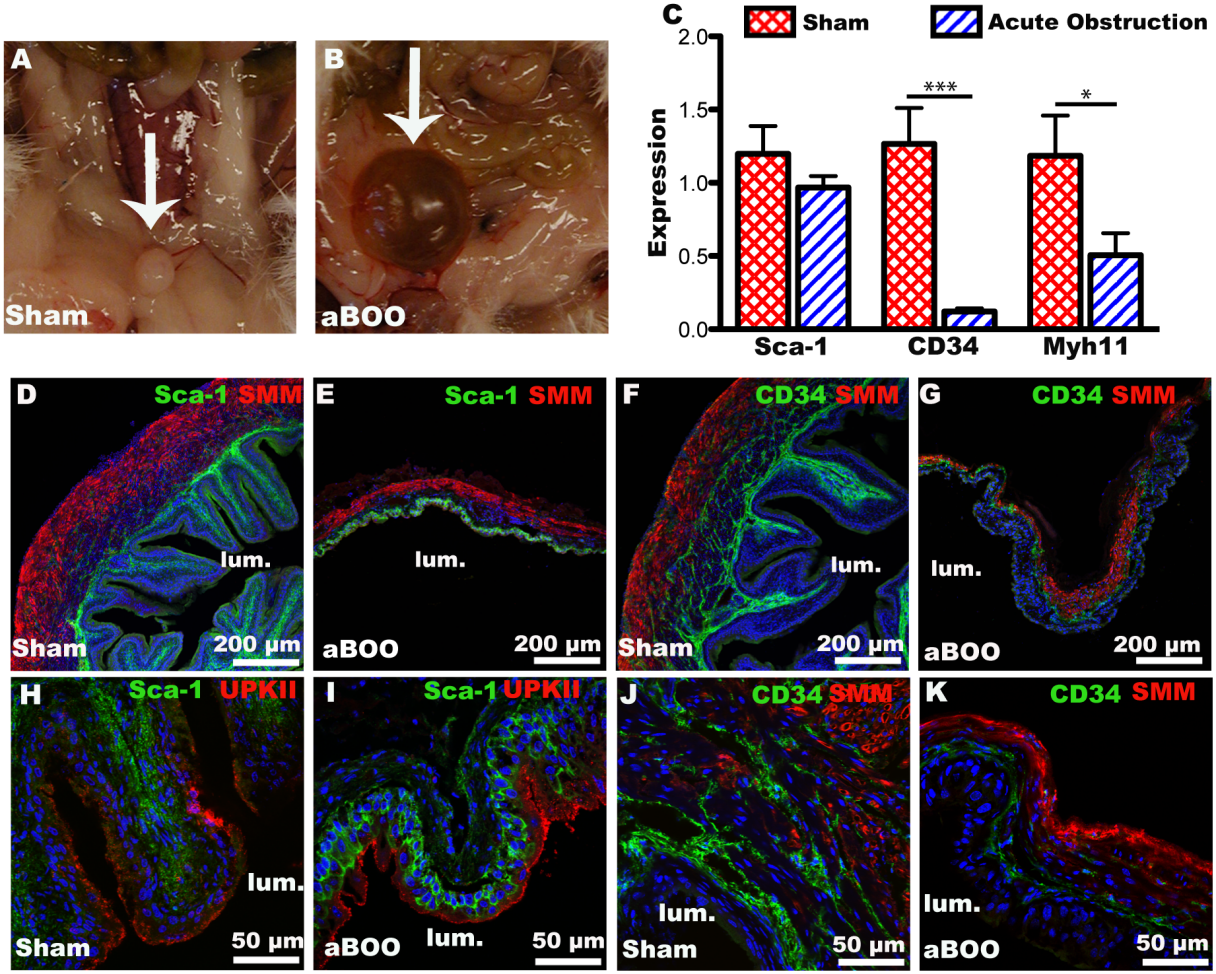
Sca-1 and CD34 expression is altered following acute bladder outlet obstruction (aBOO). (A, B) Photographs of bladders (arrows) 24h after aBOO surgery. In the sham surgery (A), the bladder has voided during anesthesia and is much smaller than the obstructed bladder (B), which is larger and distended (arrows). (C) Quantitative PCR analysis of total RNA isolated from bladders in sham surgeries (red, crosshatched bars) or after acute obstruction (blue, striped bars). Tissue was harvested 24h post surgery. *GAPDH* was used as the reference gene. (D-K) Immunofluorescence images of adult CD1 mouse bladders stained with SMM and Sca-1 (D, E), SMM and CD34 (F, G, J, K) and Uroplakin II (UPKII) and Sca-1 (H, I) in sham operated and aBOO bladders. Staining is indicated at the top of each panel. The position of the bladder lumen is indicated (lum). Scale bars are as shown. Asterisks in (C) represent significance values of P < 0.001 *** and P < 0.05 * after a Student’s T-Test.

**Fig 9:**
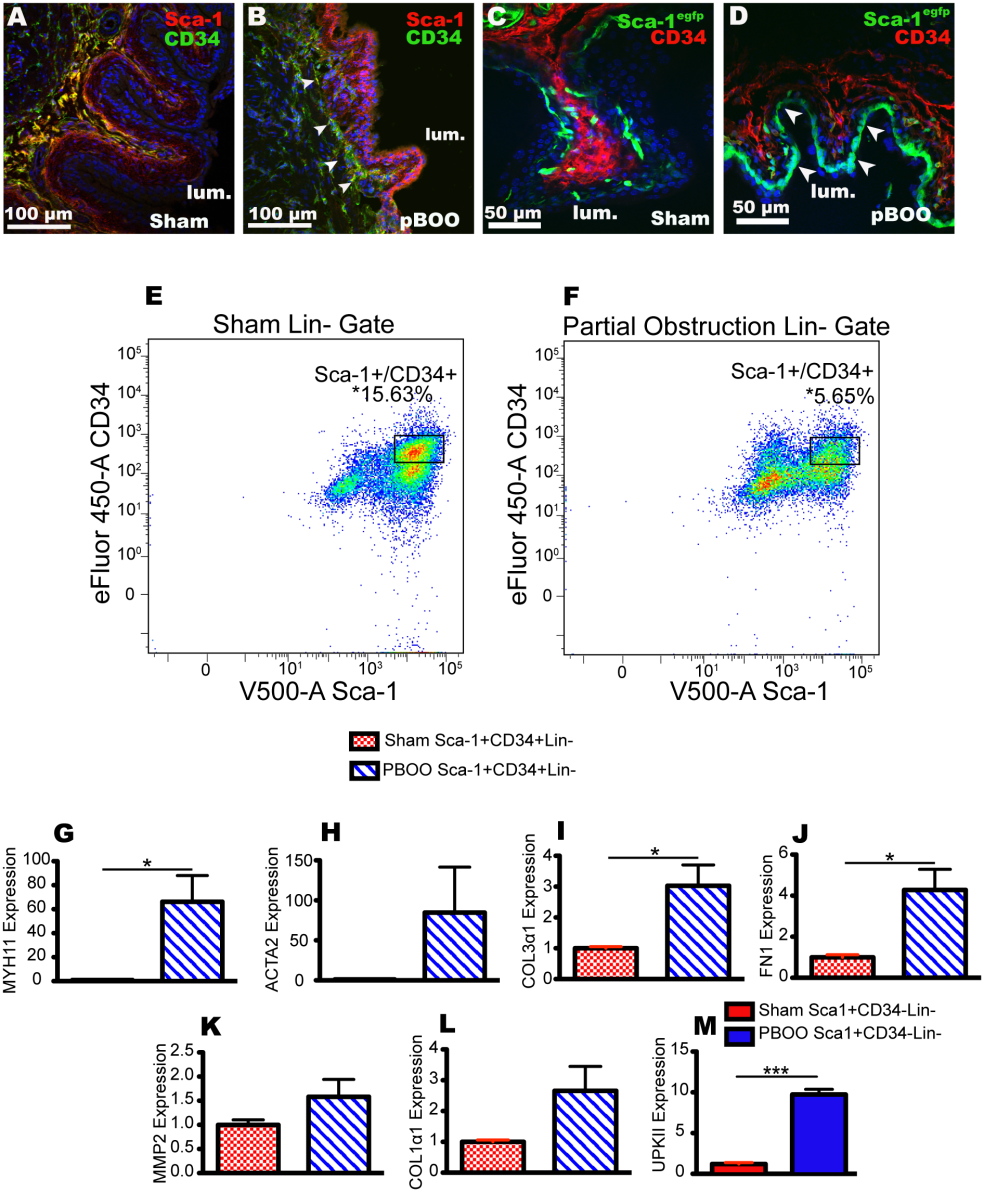
Partial bladder outlet obstruction (pBOO) alters Sca-1 expression while still maintaining a Sca-1^+^/CD34^+^ population in the stromal layer of the bladder. (A) Immunofluorescence images of Sca-1 and CD34 co-localization in a sham CD1 mouse 1wk post surgery. (B) Immunofluorescence images of Sca-1 and CD34 localization in CD1 mouse 1wk post pBOO surgery. Arrowheads (B) point to localization of Sca-1 to the urothelial layer. Arrowheads (B) point to co-localization of Sca-1 and CD34 within the stromal layer 1 week after pBOO surgery. (C, D) Immunofluorescence images of *Sca-1* ^*egfp*^ mice 1 week following sham (C) and (D) pBOO surgery. Arrowheads (D) point to localization of EGFP to the urothelial layer. (E, F) FACS analysis of two unique sorts with sham and pBOO bladder digests. Percentages reported represent percent of total living cells. Asterisks (P, Q) represent significance values P < 0.01 after a Student’s T-Test. (G-L) Quantitative PCR analysis of RNA from Sca-1^+^/CD34^+^/lin^-^ cells after sham (red cross hatched bars) and pBOO surgeries (blue striped bars). Analysis represents two technical replicates of two separate sorts; one sort had 5 pooled pBOO and 5 pooled sham CD1 mice and the other had pools of 2 pBOO and 2 sham CD1 mice. RNA was collected 7 days post pBOO surgery. Asterisks represent significance values P<0.05 * and P<0.001 *** after Student’s T-Tests. Data is normalized to sham gene expression, and GAPDH was used as the reference gene. (M) qPCR analysis of RNA of Sca-1^+^/CD34^-^/lin^-^ cells from sham surgeries (solid red bar) and pBOO surgeries (solid blue bar).

Obstruction is known to cause permanent alterations to the tissue architecture of the bladder wall. This can occur in patients even after the bladder is obstructed for a short period of time and then obstruction is surgically relieved [27]. We used two models of bladder obstruction: acute bladder outlet obstruction (aBOO), in which the bladder was completely obstructed for 24h, and partial bladder outlet obstruction (pBOO) for 7d. Partial obstruction was confirmed by post-mortem bladder size (S3 Fig) and voiding stain on paper assays (S3 Fig). Both acute and partially obstructed bladders were larger and more distended than sham operated bladders (Fig 8A and 8B, S3 Fig). Sca-1 mRNA expression in bladders from animals undergoing acute obstruction vs. sham surgery was decreased (Fig 8C). CD34 was also decreased but by a larger amount (Fig 8C). In the case of either aBOO or pBOO the bladder wall changed after obstruction (Figs 8E, 8G, 8I, 8K, 9B and 9D). Decreased amounts of total SMM mRNA expression were detected through qPCR analysis in the aBOO model (Fig 8C). Interestingly, we observed Sca-1 expression in the basal urothelium with anti-Sca-1 in aBOO (Fig 8E and 8I) and pBOO mice (Fig 9B). Similarly, a shift in *Sca1* ^*egfp*^ expression to the urothelium was detected in pBOO mice (Fig 9C and 9D arrowheads). The urothelium did not express CD34 after either aBOO or pBOO. Stromal coexpression of Sca-1 and CD34 was maintained after pBOO model of obstruction (Fig 9B, arrowheads).

We investigated if the sorting profile of Sca-1^+^/CD34^+^/lin^-^ cells changed after pBOO (hereafter we did not pursue aBOO since it is quite damaging to the bladder and mice survive only 24h after surgery). With FACS analysis, we found that the relative proportions of the Sca-1^+^/CD34^+^/lin^-^ cells changed after pBOO. In shams, this population constituted roughly 15% of living cells, while in obstructed bladders the population decreased to approximately 5% of living cells (Fig 9E and 9F).

Because we observed a change in the Sca-1^+^/CD34^+^/lin^-^ profile after pBOO, we next analyzed gene expression in this population using qPCR (Fig 9G–9M). We tested if smooth muscle genes were altered in response to pBOO. In sham operated mice neither *Myh11* or *ACTA2* were detected in the Sca-1^+^/CD34^+^/lin^-^ population, but both genes were increased in Sca-1^+^/CD34^+^/lin^-^ cells after pBOO (Fig 9G and 9H). Sca-1^-^/CD34^-^/lin^-^ cells also expressed *Myh11* and *ACTA2* in obstructed, although SMM was decreased when compared to the sham (S3 Fig). We observed an approximate 10-fold increase in the expression of *uroplakin II* in Sca-1^+^CD34^-^lin^-^ cells (Fig 9M), consistent with our observation that Sca-1 expression is increased in the urothelium after obstruction.

Since obstruction results in a high-pressure bladder that has difficulty emptying, the bladder wall responds by becoming more rigid. This usually involves the production of fibrotic matrices typically marked by the accumulation of collagen fibers. We tested if FACS sorted cells increased their expression of fibrosis associated genes after pBOO. Within the Sca-1^+^/CD34^+^/lin^-^ population, we observed significant increased expression of collagen type 3, alpha 1 (COL3α1) and in fibronectin 1 (FN1) between the sham and pBOO model (Fig 9I and 9J). Matrix metalloproteinase 2 (MMP2) and collagen type 1, alpha 1 (*Col1* ***α*** *1*) were increased but this difference was not statistically significant (Fig 9K and 9L). Thus, obstructive injury reduced the number of Sca-1^+^/CD34^+^/lin^-^ mesenchymal stems cells and caused these cells to express both smooth muscle cytoskeletal genes and genes that are increased in fibrosis.

## Discussion

Here we present evidence that the stromal layer of the bladder contains a population of Sca-1^+^/CD34^+^/lin^*-*^ mesenchymal stem cells. This population resembles Sca-1^+^/CD34^+^/lin^*-*^ cells found in the adventitia of arteries and in lung [8,9,14]. Like these other Sca-1^+^/CD34^+^/lin^*-*^ cell-types, mouse bladder mesenchymal stem cells can differentiate into smooth muscle-like cells *ex-vivo*. Together with urothelial progenitor cells [6], we believe that there are two distinct progenitor lineages in the adult mouse bladder. It is reported that the adult rodent bladder can regenerate *de novo* [28,29] and bladder augmentations constructed from extracellular matrix become cellularized [30], so it is likely that intrinsic stem-like bladder cells contribute to the re-growth of the bladder after injury.

We do not yet know the source of stromal Sca-1^+^/CD34^+^/lin^*-*^ mesenchymal stem cells. One possibility is that these cells are derived from circulating cells originating from circulating hematopoietic stem cells (HSC). However, Sca-1^+^/CD34^+^ cells are present in the stroma of the P01, suggesting that these cells are derived from bladder development and not from HSCs. Studies have tested if labeled donor cells contributed to the adult bladder after bone marrow replacement and these studies found that a small number of labeled cells arrived in the stromal layer of the bladder [5]. We believe that this small number cannot account for the total number of Sca-1^+^/CD34^+^/*lin* ^*-*^ cells we see in the adult mouse bladder, but our experiments do not rule out the possibility that the stromal mesenchymal cells we observe are derived from embryonic HSCs.

Our results indicate that the mechanism of colonizing the stroma with Sca-1^+^/CD34^+^/lin^-^ cells occurs by P01. This means that the original population of stromal mesenchymal stem cells is largely derived during embryonic development. This time was later than observed with adventitial Sca-1^+^/CD34^+^/lin^-^ cells, which were observed first in the perivascular space of the great arteries as early as E15.5 [14]. We saw that CD34 was expressed in a large number of cells within the bladder wall including areas where smooth muscle was differentiating. As development continued the CD34+ population appeared to define the bladder stroma before Sca-1 protein was expressed there. However, once formed by E18.5, the stroma began to express Sca-1 and this increased so that two days later, the pattern of expression of Sca-1 reflected the pattern we observed in adult bladders. Since Sca-1^+^/CD34^+^ cells are found in the bladder stroma at birth, we believe that the stromal MSCs we have defined represent an intrinsic or resident population of progenitor cells.

We believe that the stromal layer could create an appropriate environment or niche for mesenchymal stem cells in the bladder. This is supported by the findings of Woo et al., (2011) who observed that non-bladder derived MSCs injected into the tail vein were recruited to the stroma after partial bladder outlet obstruction. In addition, bone marrow replacement experiments demonstrated that circulating stem cells also target the bladder stroma [4,5]. The stroma is an extracellular matrix rich, loose mesenchyme similar to other stem niches such as the hematopoietic [31], hair follicle, intestinal and vascular adventitial niches. Thus, among other things, its role may be to provide an environment that is suitable for both resident stem and circulating HSCs that are targeted to the bladder.

It is likely that the adult mouse bladder contains more than one population of progenitor cells. Shin and colleagues (2011) described a population of Shh expressing basal urothelial cells that have progenitor cell properties [6]. These cells proliferated and repopulated the urothelium of bladders challenged with a pathogenic strain of *Escherichia coli*. FACS sorted cells formed self-renewing organoids in culture and lineage tracing demonstrated that these cells could regenerate all cell types of the urothelium after injury. Interestingly, their findings demonstrated an increase in urothelial expression of Shh and an increase in stromal *Wnt* and *FGF* genes after infection. Shh signal originating in the basal epithelial layer of the urothelium was proposed to be received by cells in the stroma, leading to downstream expression of *Gli1*, *Patched*, and *Axin2*. Bladders from *Gli1* mutant mice had decreased cell proliferation in the stroma after infection and this could be modulated by pharmacologic reduction or augmentation of *Wnt* signaling. These findings indicate that, after injury, the urothelium establishes a chemical-based communication with the stroma to regulate cell-proliferation. These signals may be a way that the stroma can respond to injury and promote cell growth therein.

While resident MSCs have been identified in many organs, it is not clear what their functions are within a normal, healthy organ. Even transplantation of sub cultured MSCs into hosts only reveals the potential of such cells in an artificial *in vivo* context. The true experimental paradigm for understanding the role of these cells is *in vivo* lineage analysis and this is particularly challenging. For example, if resident MSCs slowly contributed to an organ over the lifetime of an animal, then a lineage tracing experiment would involve investigation for a span of months or years. A few groups have attempted such analyses, but in most cases the true relevance of resident stem cells can only speculated upon. In the case of bladder, stromal MSCs, the appropriate Cre driver that could be used in lineage tracing experiments is still unresolved. The *Gli1* ^*CreERT2*^ mouse line was used to investigate the role of perivascular MSCs in mice [32] and we had considered that this line might be suitable for labeling bladder Sca-1^+^/CD34^+^/lin^-^ cells. However, we observed that the *Gli1* ^*CreERT2*^ gene promoted recombination in only about 10% of Sca-1^+^/CD34^+^/lin^-^ cells and in numerous cells outside the stroma. Thus, it became clear that better Cre models will need to be developed before the role of bladder Sca-1^+^/CD34^+^/lin^-^ MSCs in the bladder can be studied *in vivo*. With respect to the role that Sca-1^+^/CD34^+^/lin^-^ cells play in an obstructed bladder, the observed upregulation in expression of genes such as Col3α1 and FN1 might provide insight into this population’s contribution to the fibrosis that often accompanies bladder obstruction.

Another unexpected finding in our study was the pattern of expression of the *Sca-1* ^*egfp*^ gene. First, it was expressed in the developing urothelium prior to our ability to detect the protein with the Sca-1 antiserum. In adults, *Sca-1* ^*egfp*^ was observed in stromal and in isolated cells in the detrusor muscle. However, after obstructive injury, *Sca-1* ^*egfp*^ was re-expressed in the urothelium. In this case, urothelial localization was detected by the Sca-1 antiserum. This indicates that obstructive uropathy restores the embryonic pattern of expression of Sca-1, which may be a response reflecting how the urothelium needs to adapt after the injury. Activation of the “fetal gene program” has been observed in other pathologies, such as congestive heart failure [33]. The reasons why embryonically expressed genes are re-expressed is not completely understood, but in some cases the presence of these genes can be used as disease markers. Sca-1 itself would not currently be useful in patients because there is no known human Sca-1 homolog to date. However, urothelial Sca-1 in mice may indicate that other fetal genes are expressed after obstruction and these could be used as indicators of remodeling before other symptomatic changes occur.

## Supporting Information

S1 Fig (A-E) Unstained cells from CD1 bladder digests for gating and compensation controls. (F) Cells from CD1 bladder digests stained singly for each fluorochrome used during FACS sorting to aid in compensation and gating.
(TIF)

S2 Fig (A) qPCR analysis of the indicated FACS sorted cell populations for expression of collagen type I, alpha I (*Col1* ***α*** *1*). Results are averages from 4 separate sorts with 3–5 pooled CD1 mice. (B) qPCR analysis of the indicated FACS sorted cell populations for expression of keratin 5 (KRT5). Results are averages from 3 separate sorts each with 3–4 pooled CD1 mice. (C) Representative colony forming units (CFUs) grown on 10cm tissue culture plates for 2w and stained with crystal violet. Arrowheads point to small number of colonies on plates seeded with Sca-1^+^/CD34^-^/lin^-^ and total live cells. Sorting profile is indicated below each image. (D) Sca-1^+^/CD34^+^/lin^-^ cells grown in α-MEM and stained with alizarin red for calcium deposition. (E) Sca-1^-^/CD34^-^/lin^-^ cells grown in osteogenic induction medium and stained with alizarin red for calcium deposition. (F) Sca-1^+^/CD34^+^/lin^-^ cells grown in α-MEM and stained with Oil Red O to assess lipid droplet formation. (G) Sca-1^-^/CD34^-^/lin^-^ cells grown in adipogenic induction medium and stained with Oil Red O to assess lipid droplet formation. (H) Sca-1^+^/CD34^+^/lin^-^ cells grown in α-MEM and stained for alkaline phosphatase. (I) Cross section of a mouse ear stained with toluidine blue demonstrating *in vivo* staining of cartilage.
(TIF)

S3 Fig (A, B) Immunofluorescence images of Sca-1^egfp^ bladders showing EGFP in the stroma (A) and detrusor muscle (B). (C, D) Photographs of bladders excised 7 days post sham and pBOO surgery. (E, F) Voiding stain on paper (VSOP) of sham and pBOO CD1 mice 7 days post operation to assess obstruction. As indicated by the number and size of the stains on the pBOO mouse VSOP (F), voiding frequency was higher (arrowheads). (G) qPCR analysis of sham Sca-1^-^/CD34^-^/lin^-^ (red hatched bar) vs. pBOO Sca-1^-^/CD34^-^/lin^-^ (blue hatched bar) cells for expression of smooth muscle myosin (*Myh11*). Results are averages from two separate sorts each with 5 or 2 CD1 mice pooled in each group. Results are normalized to pBOO expression of SMM.
(TIF)

S1 File Reagents used in this study. **Table A,** Primary antibodies used in confocal fluorescent microscopy. **Table B,** Secondary antibodies used for confocal fluorescent microscopy. **Table C**, Conjugated antibodies used for flow cytometry and FACS. **Table D,** Primers used in qPCR analysis.
(DOCX)
