# Supplementary figures and images for: The Murine Bladder Supports a Population of Stromal Sca-1+/CD34+/lin- Mesenchymal Stem Cells
Source: PLoS One. 2015 Nov 5;10(11):e0141437. doi: 10.1371/journal.pone.0141437 (PMC4634995; doi:10.1371/journal.pone.0141437)

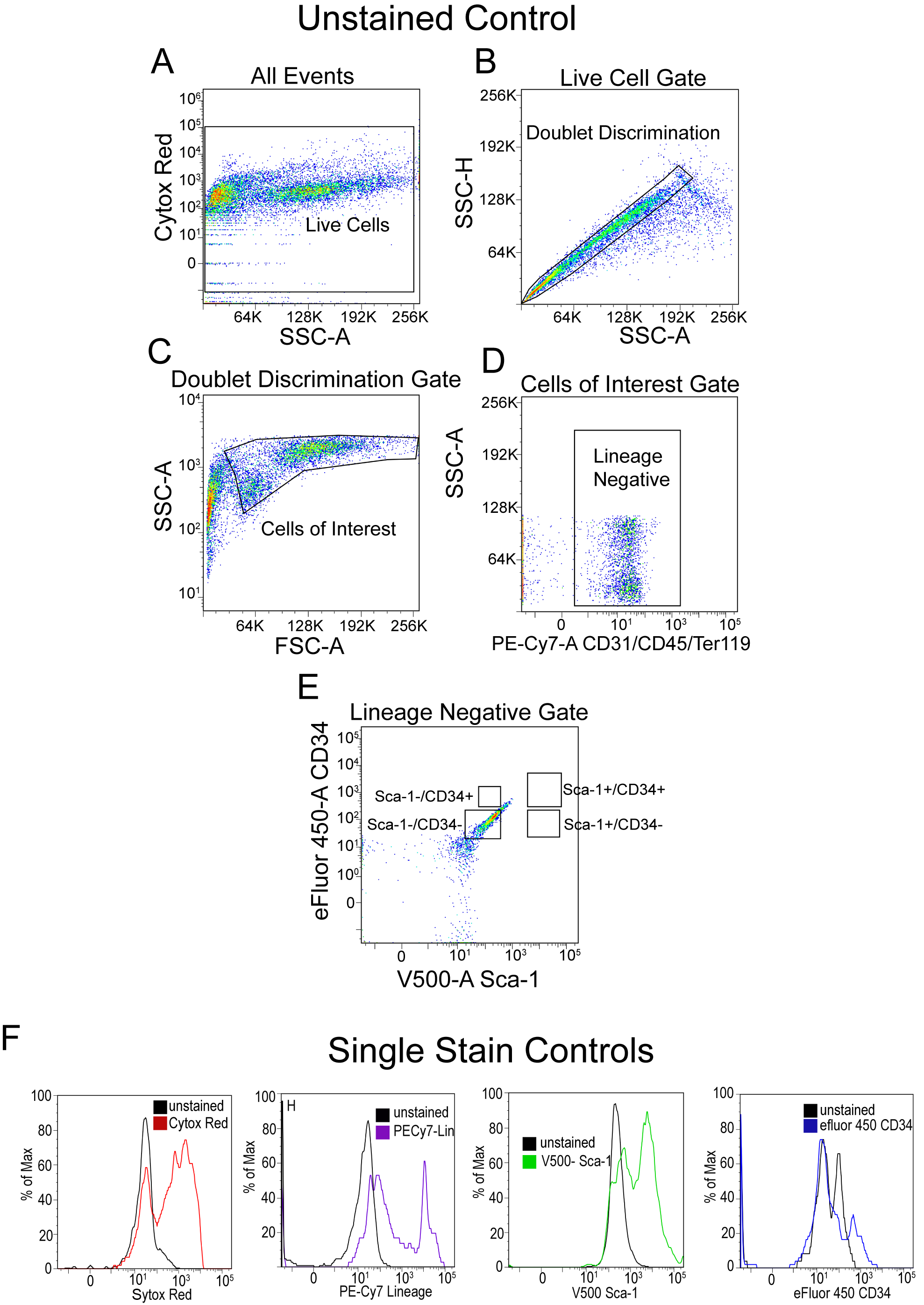

Supplement: S1 Fig — (F) Cells from CD1 bladder digests stained singly for each fluorochrome used during FACS sorting to aid in compensation and gating. (TIF) [file pone.0141437.s001.tif]

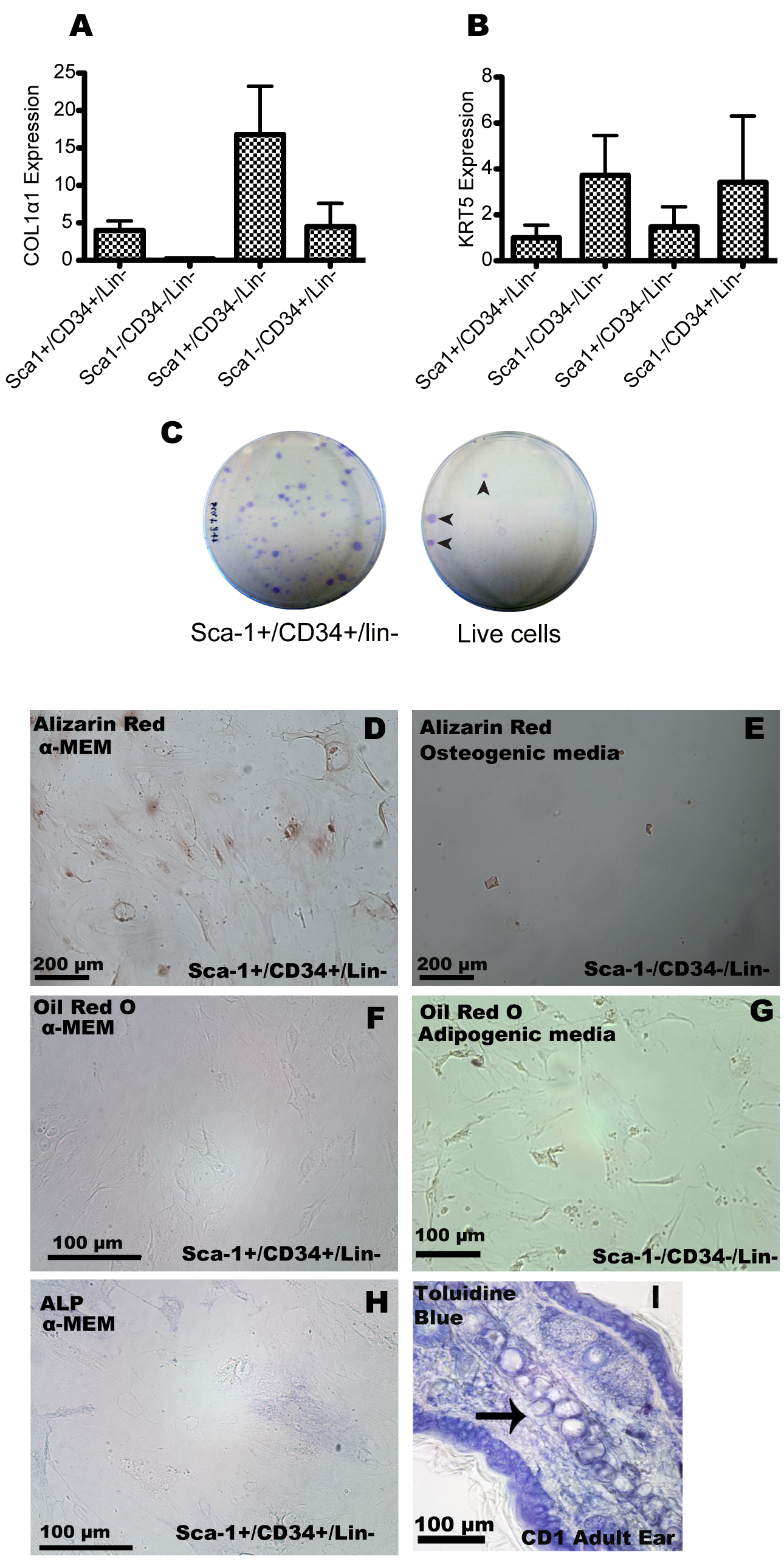

Supplement: S2 Fig — (A) qPCR analysis of the indicated FACS sorted cell populations for expression of collagen type I, alpha I (Col1 α 1). Results are averages from 4 separate sorts with 3–5 pooled CD1 mice. (B) qPCR analysis of the indicated FACS sorted cell populations for expression of keratin 5 (KRT5). Results are averages from 3 separate sorts each with 3–4 pooled CD1 mice. (C) Representative colony forming units (CFUs) grown on 10cm tissue culture plates for 2w and stained with crystal violet. Arrowheads point to small number of colonies on plates seeded with Sca-1+/CD34-/lin- and total live cells. Sorting profile is indicated below each image. (D) Sca-1+/CD34+/lin- cells grown in α-MEM and stained with alizarin red for calcium deposition. (E) Sca-1-/CD34-/lin- cells grown in osteogenic induction medium and stained with alizarin red for calcium deposition. (F) Sca-1+/CD34+/lin- cells grown in α-MEM and stained with Oil Red O to assess lipid droplet formation. (G) Sca-1-/CD34-/lin- cells grown in adipogenic induction medium and stained with Oil Red O to assess lipid droplet formation. (H) Sca-1+/CD34+/lin- cells grown in α-MEM and stained for alkaline phosphatase. (I) Cross section of a mouse ear stained with toluidine blue demonstrating in vivo staining of cartilage. (TIF) [file pone.0141437.s002.tif]

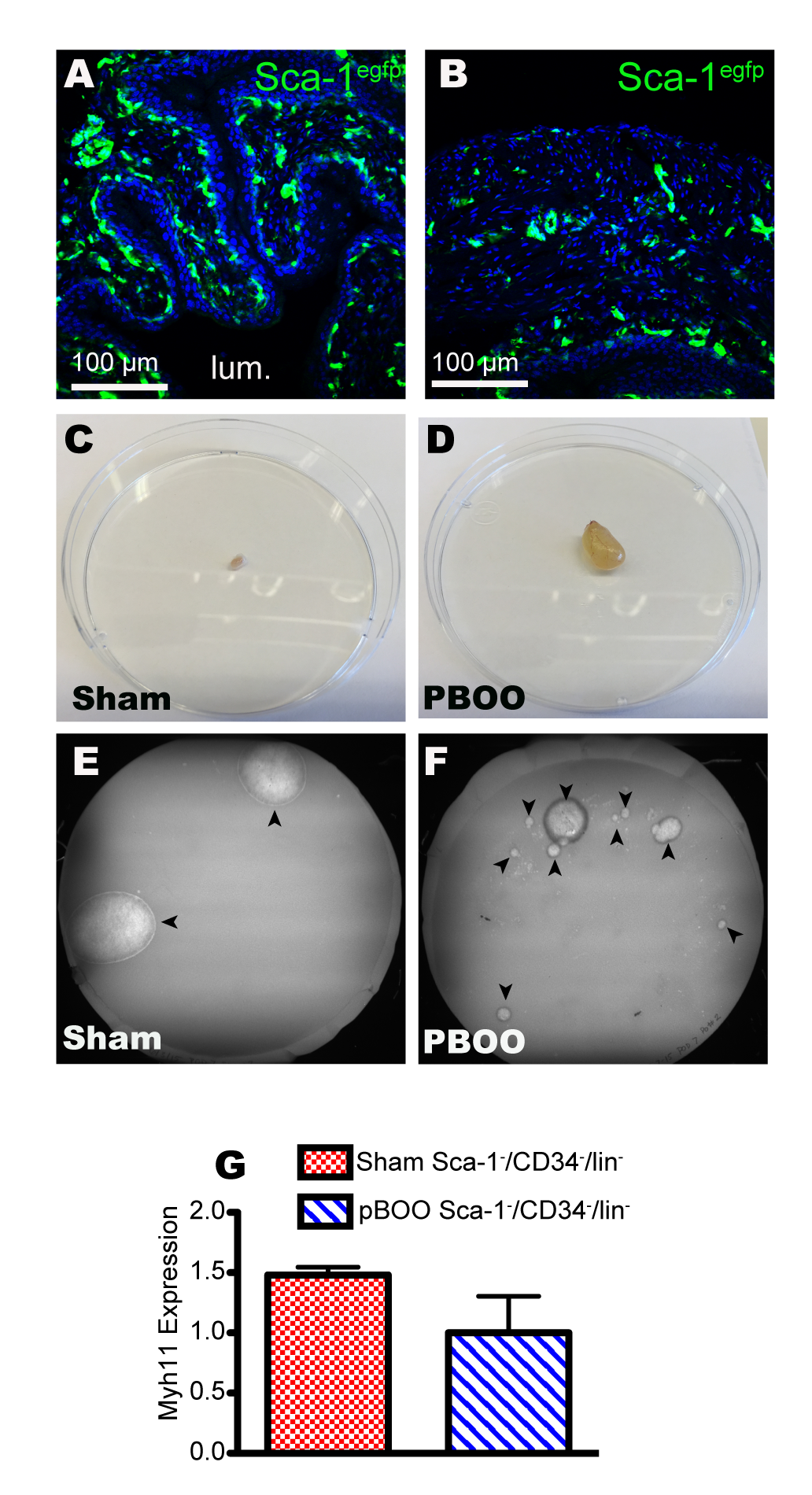

Supplement: S3 Fig — (A, B) Immunofluorescence images of Sca-1egfp bladders showing EGFP in the stroma (A) and detrusor muscle (B). (C, D) Photographs of bladders excised 7 days post sham and pBOO surgery. (E, F) Voiding stain on paper (VSOP) of sham and pBOO CD1 mice 7 days post operation to assess obstruction. As indicated by the number and size of the stains on the pBOO mouse VSOP (F), voiding frequency was higher (arrowheads). (G) qPCR analysis of sham Sca-1-/CD34-/lin- (red hatched bar) vs. pBOO Sca-1-/CD34-/lin- (blue hatched bar) cells for expression of smooth muscle myosin (Myh11). Results are averages from two separate sorts each with 5 or 2 CD1 mice pooled in each group. Results are normalized to pBOO expression of SMM. (TIF) [file pone.0141437.s003.tif]
