# Supplementary material for: The Murine Bladder Supports a Population of Stromal Sca-1+/CD34+/lin- Mesenchymal Stem Cells
Source: PLoS One. 2015 Nov 5;10(11):e0141437. doi: 10.1371/journal.pone.0141437 (PMC4634995; doi:10.1371/journal.pone.0141437)
Supplement: S1 File — Table A, Primary antibodies used in confocal fluorescent microscopy. Table B, Secondary antibodies used for confocal fluorescent microscopy. Table C, Conjugated antibodies used for flow cytometry and FACS. Table D, Primers used in qPCR analysis. (DOCX) [file pone.0141437.s004.docx]

**S1 File**

| **Primary Antibody Name** | **Common Name** | **Company** | **Reference Number** |
| --- | --- | --- | --- |
| Rab. mAb to Calponin | Calponin | Abcam | Ab46794 |
| Rat anti-mouse CD34 | CD34 | eBioscience | 14-0341-81 |
| Rat anti-mouse Ly6A/E | Sca-1 | BD Pharmingen | 557403 |
| Rabbit anti-SRF (G-20) | Serum response factor | Santa Cruz Biotechnology | Sc-335 |
| Rabbit anti-smooth muscle myosin IgG | Smooth muscle myosin | Biomedical Technologies | BT-562 |
| Rabbit anti-sonic hedgehog | Sonic hedgehog | Santa Cruz Biotechnology | Sc-9024 |
| Goat anti-UP2 | Uroplakin | Santa Cruz Biotechnology | Sc-15178 |

**Table A**. Primary antibodies used in confocal fluorescent microscopy.

| **Secondary Antibody Name** | **Company** | **Reference Number** |
| --- | --- | --- |
| Alexa-Fluor 488 donkey anti-Rat IgG | Life Technologies | A21208 |
| Alexa-Fluor 647 goat anti-Rat IgG | Life Technologies | A21247 |
| Alexa-Fluor 546 donkey anti-rabbit IgG | Life Technologies | A10040 |
| Alexa-Fluor 633 goat anti-rabbit IgG | Life Technologies | A21070 |
| Alexa-Fluor 546 rabbit anti-goat IgG | Life Technologies | A11056 |

**Table B**. Secondary antibodies used for confocal fluorescent microscopy.

| **Antibody Name** | **Fluorochrome** | **Company** | **Reference number** |
| --- | --- | --- | --- |
| Anti-mouse Ly6A/E | PE | eBioscience | 12-5981-81 |
| Anti-mouse Ly6A/E | PerCP-Cyanine 5.5 | eBioscience | 45-5981-80 |
| Anti-mouse Ly6A/E | V500 | BD Horizon | 561229 |
| Anti-mouse CD34 | eFluor450 | eBioscience | 48-0341-80 |
| Anti-mouse CD34 | FITC | eBioscience | 11-0341-81 |
| Anti-mouse CD31 | PE Cyanine7 | eBioscience | 25-0311-81 |
| Anti-mouse CD31 | FITC | eBioscience | 11-0311-81 |
| Anti-mouse CD45 | PE Cyanine7 | eBioscience | 25-0451-81 |
| Anti-mouse CD45 | FITC | eBioscience | 11-0451-81 |
| Anti-mouse Ter119 | PE Cyanine7 | eBioscience | 25-5921-81 |
| Anti-mouse Ter 119 | FITC | eBioscience | 11-5921-81 |

**Table C**. Conjugated antibodies used for flow cytometry and FACS.

| **Gene Name** | **Common Name** | **Company** | **Reference Number** |
| --- | --- | --- | --- |
| GAPDH | GAPDH | Life Technologies | Mm99999915_g1 |
| Ly6a | Sca-1 | Life Technologies | Mm04337234_mH |
| CD34 | CD34 | Life Technologies | Mm00519283_m1 |
| MYH11 | Smooth muscle myosin | Life Technologies | Mm00443013_m1 |
| ACTA2 | Alpha Actin | Life Technologies | Mm00725412_s1 |
| SRF | Serum response factor | Life Technologies | Mm00491032_m1 |
| Vimentin | Vimentin | Life Technologies | Mm01333430_m1 |
| FN1 | Fibronectin 1 | Life Technologies | Mm01256744_m1 |
| COL3A1 | Collagen Type III, Alpha 1 | Life Technologies | Mm01254476_m1 |
| COL1A1 | Collagen Type I, Alpha 1 | Life Technologies | Mm00801666_g1 |
| UPKII | Uroplakin 2 | Life Technologies | Mm04207773_g1 |

**Table D**. Primers used in qPCR analysis.
